# Time-resolved compositional and dynamics analysis of biofilm maturation and dispersal via solid-state NMR spectroscopy

**DOI:** 10.1038/s41522-025-00655-4

**Published:** 2025-01-29

**Authors:** Yi Xue, Xue Kang

**Affiliations:** Institute of Drug Discovery Technology, Ningbo University, Ningbo, 315211 Zhejiang China

**Keywords:** Bacteria, Biofilms

## Abstract

Dispersal plays a crucial role in the development and ecology of biofilms. While extensive studies focused on elucidating the molecular mechanisms governing this process, few have characterized the associated temporal changes in composition and structure. Here, we employed solid-state nuclear magnetic resonance (NMR) techniques to achieve time-resolved characterization of *Bacillus subtilis* biofilms over a 5-day period. The mature biofilm, established within 48 h, undergoes significant degradation in following 72 h. The steepest decline of proteins precedes that of exopolysaccharides, likely reflecting their distinct spatial distribution. Exopolysaccharide sugar units display clustered temporal patterns, suggesting the presence of distinct polysaccharide types. A sharp rise in aliphatic carbon signals on day 4 probably corresponds to a surge in biosurfactant production. Different dynamic regimes respond differently to dispersal: the mobile domain exhibits increased rigidity, while the rigid domain remains stable. These findings provide novel insights and perspectives on the complex process of biofilm dispersal.

## Introduction

Biofilms are sophisticated, multicellular communities of microorganisms that adhere to surfaces and encase themselves within a self-produced matrix of extracellular polymeric substances (EPS)^1–4^. This protective matrix shields the cells from external threats and creates a unique microenvironment that enhances their survival in hostile conditions^5^. The life cycle of a biofilm is a dynamic and highly regulated process, regulated by intricate genetic pathways and influenced by various environmental cues and microbial interactions^6–10^. This cycle is described as several phenotypically distinct yet sequentially interconnected stages: initial attachment, microcolony formation, maturation, and dispersal^11^. Throughout this cycle, each stage is marked by unique patterns of genetic activity and protein synthesis, reflecting the changing needs and behaviors of the microbial community^12–14^.

Extensive research has focused on identifying key intrinsic and external regulators, effectors, and molecular mechanisms involved in biofilm development^6,7,15^. For instance, the role of cyclic-di-GMP as a central second messenger has been extensively characterized across various bacterial species^16–20^; Quorum sensing closely coordinates biofilm formation and dispersal by modulating gene expression in response to changes in population density^15,21,22^; A plethora of transcriptional factors and small RNAs have been identified that specifically regulate biofilm-associated gene expression^23–26^; Moreover, external factors such as nutrient availability, pH, and temperature have also been thoroughly studied to understand their impacts on the biofilm dynamics^26–28^.

Alongside these molecular studies, the structural dynamics of biofilm development have also been explored using advanced imaging and microscopic techniques. For example, fluorescent reporters and staining techniques have been used in conjunction with confocal laser scanning microscopy to visualize the spatial distribution of specific biofilm components, such as cells and extracellular polymeric substances (EPS), over time^29,30^. Moreover, the spatiotemporal effects of various environmental conditions, including different nutrient sources, antimicrobial treatments, and metal ions, can be effectively monitored and assessed on biofilm structure and composition^31,32^. While these techniques have provided valuable qualitative insights into the spatial and temporal distribution of certain biofilm constituents, precise and comprehensive quantitative analysis on the changes in the abundance of various biofilm components over time remain scarce due to the universal technical challenges for studying complex bio-solid samples. The knowledge scarcity impedes the accurate characterization and description of the dynamic process and elucidation of the underlying mechanisms that drive these nuanced changes.

The Gram-positive bacterium *Bacillus subtilis* (*B. subtilis*) serves as a model organism for biofilm research. Its biofilm matrix consists primarily of exopolysaccharides, TasA protein fibers, and BslA hydrophobins^33–38^. These components are synthesized under the control of the *epsA*-*O* operon, *tapA*-*sipW*-*tasA* operon, and *bslA* gene, respectively. The repressor proteins AbrB and SinR regulate these genetic elements^39–41^. While the formation of *B. subtilis* biofilms is well characterized, the process of biofilm dispersal remains poorly understood. Most recent studies still continued to focus on investigating various factors that influence biofilm dispersal, including external conditions^42^, signaling molecules^43^, and intrinsic regulatory pathways^44^. Quantitative data describing this process are still lacking and pose a significant knowledge gap.

**Fig. 1:**
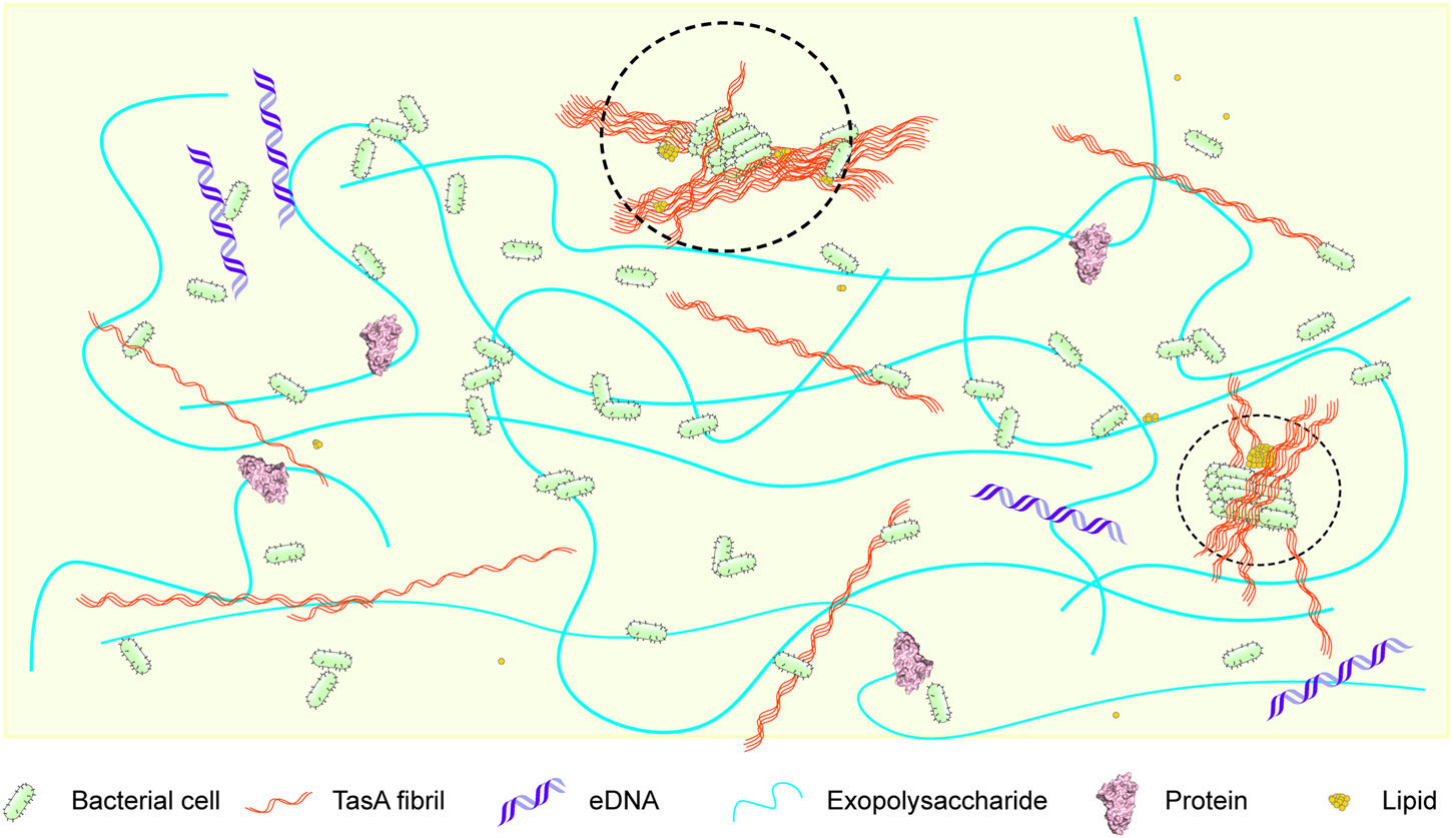
Conceptual model of the *B. subtilis* biofilm based on ssNMR characterization from our previous study^45^. Dashed line circles highlight the minor rigid phase (solid-like) present within biofilm, including bacterial cells, TasA fibers, and lipids. The majority of biofilm exhibits significant molecular mobility, consistent with its macroscopic viscoelastic properties. The predominant mobile phase (liquid-like) consists of exopolysaccharides, proteins, TasA, eDNA, bacterial cells, and lipids. This conceptual representation of bacterial cell distribution emphasizes the contrast between rigid and mobile phases. While the figure accurately represents the molecular compositions across different dynamic regimes, the illustrations are not drawn to the actual scale of individual molecules.

Our previous research has laid the groundwork for applying advanced solid-state NMR (ssNMR) techniques to the in situ characterization of biofilms, specifically using *B. subtilis*^45^. We have elucidated distinct dynamics regimes within the mature biofilm from an NMR perspective, identifying that 90% of the components in the dominant mobile phase (liquid-like behaviors) and 10% in the minor rigid phase (solid-like characteristics) (Fig. 1). With high-resolution ssNMR spectra, quantitative analysis was performed not only on the intact samples but also with an unprecedented detail, revealing the compositions at various hierarchical levels. Building upon the well-established methodology and acquired compositional information, we are well-positioned to expand our investigations into the dynamic nature of biofilms by incorporating the time dimension.

In this study, we thoroughly examined the development of *B. subtilis* biofilm in a static medium over a 5-day period. Through the non-destructive, quantitative assessment of structural components and the unique analysis of distinct dynamic regimes within the intact biofilm samples, we address a critical knowledge gap in biofilm dynamics from the perspective of compositional and structural evolution. Meticulous analysis of temporal profiles not only elucidates apparent compositional shifts and events, but also unveils hidden clustered patterns among diverse monosaccharide components within the complex exopolysaccharides. The molecular dynamics evolution of different structural components, when integrated with their temporal abundance profiles, yields profound insights into their spatial organization and functional mechanisms within the biofilm matrix. Overall, our pioneering work not only advances the fundamental understanding of biofilm development process but also establish a quantitative framework for evaluating biofilm dynamics under various conditions.

## Methods

### Preparation of ^13^C-labeled biofilm samples

*B. subtilis* (strain NCIB3610) was grown in Luria–Bertani (LB) broth at 37 °C with a shaking speed of 225 rpm until reaching mid log phase. The culture was then diluted 1:1000 in 6 mL of the modified MSgg medium^46^ and incubated statically at 30 °C for varying durations before harvesting. Isotope labeling was achieved by substituting glycerol, the carbon source in the medium, with ^13^C-labeled glycerol (Cambridge Isotope Laboratories, Tewksbury)^45^.

The biofilm samples, each prepared in duplicate (Supplementary Fig. 1), were harvested at 1, 2, 3, 4, and 5 day(s) post inoculation. The collected samples were washed three times with distilled water by gentle pipetting. The resulting supernatant was collected and analyzed by 1D ^1^H spectrum (Supplementary Fig. 2) to check if the washing step appreciably dissolved any loosely bound biofilm components. Approximately 30 mg of each biofilm sample was packed into a 3.2-mm magic-angle spinning (MAS) rotor by multiple centrifugation steps (3000 × *g*, 30 s each) for ssNMR data collection. After each centrifugation step, any pooled water at the top was carefully removed using pipette tips. Before sealing the rotor, residual water was thoroughly cleaned to ensure safe high-speed spinning during the experiment. A high-speed test spin at 13.5 kHz was conducted for each sample for 30 min to ensure no residual liquid remained within the rotor. The liquid medium was centrifuged at 12,000 × *g* for 20 min to remove insoluble material and then subjected to solution NMR analysis. The reproducibility of replicated samples was examined and confirmed (Supplementary Figs. 1 and 3, Supplementary Tables 2 and 3). Subsequent data analysis was conducted using a single dataset (batch-2 samples).

### Solution NMR experiments for medium analysis

5% D_2_O (with 0.01% 4,4-dimethyl-4-silapentane-1-sulfonic acid (DSS)) was added to the medium sample as the locking solvent. 1D ^13^C spectra and 2D ^1^H–^13^C heteronuclear single quantum coherence (HSQC) spectra were collected on a Bruker Avance Neo 600 MHz spectrometer equipped with a 5 mm ^1^H/^31^P/^13^C/^15^N cryoprobe at 295 K. The detailed experimental acquisition parameters are listed in Supplementary Table 15.

**Fig. 2:**
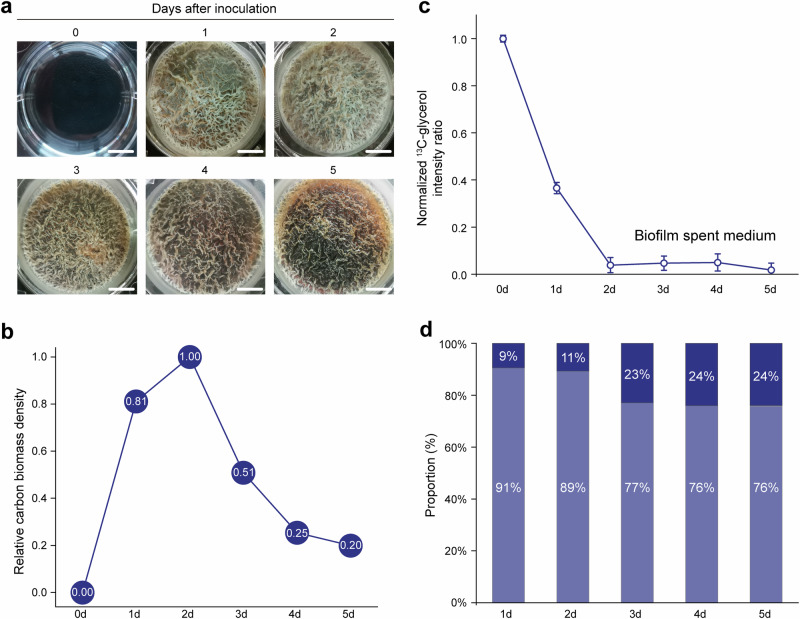
Biofilm development over a 5-day period. **a** Strain 3610 was statically grown at 30 °C in a 6-well plate to form pellicles. Top views of the pellicles are shown, with an uninoculated well included as a reference. Scale bars represent 10 mm. **b** Relative carbon biomass density during biofilm development. The Day-2 sample is set as the reference value of 1.0. **c** ^13^C-glycerol content in the culture medium during biofilm development. **d** Proportion of biomass in mobile phase (light blue) and rigid phase (dark blue) during biofilm development.

In the 1D ^13^C spectra, glycerol exhibits characteristic signals at 65.187 and 72.775 ppm. Glycerol consumption was monitored and quantified by comparing the intensity of the 65.2 ppm peak, as detailed in Supplementary Table 1 and Fig. 2c.

Carbohydrate assignments in the culture medium HSQC spectra were informed by ssNMR measurements of the biofilm samples. By cross-referencing with the anomeric carbon and proton signals identified in the biofilm through ssNMR 2D ^13^C–^1^H INEPT spectra, we observed that the majority of carbohydrate compositions identified in the biofilm are also present in the medium, confirming the continuous release of biofilm carbohydrates into the medium (Supplementary Fig. 6, Supplementary Tables 6 and 7).

It’s important to note that the HSQC method is not strictly quantitative. However, when spectra are acquired under identical experimental conditions and T_2_ variations among different carbohydrates are neglected, HSQC can provide valuable semi-quantitative and comparative insights into compositional differences among samples. Following this principle, the integration of assigned anomeric carbon signals was performed and compared (Supplementary Table 11, Fig. 5e).

### SsNMR experiments for biofilm analysis

All ssNMR experiments were conducted on a Bruker Avance Neo 800 MHz spectrometer equipped with a 3.2 mm ^1^H/^13^C/^15^N E-free MAS probe. The probe temperature was set at 275 K. The ^13^C chemical shifts in ssNMR experiments were externally referenced to the adamantane CH_2_ signal at 38.48 ppm on the tetramethylsilane (TMS) scale. Typical radiofrequency strength was 50–83.3 kHz for ^1^H and 62.5 kHz for ^13^C. The acquisition parameters and details are listed in Supplementary Table 15.

A series of one-dimensional (1D) ^13^C NMR spectra were acquired using distinct polarization methods to analyze biofilm composition. Quantitative measurement of the total biofilm carbon content was obtained using direct polarization (DP) with a 15 s recycle delay, accommodating the longest spin-lattice relaxation times (~2.6 s) of biomolecules within the biofilm. Mobile components (liquid-like, with tumbling rate ≥10^–9^ sec^–1^)^47^ were selectively detected by DP with a shorter 2 s recycle delay. Rigid components (solid-like, with tumbling rate ≤10^–5^ sec^–1^)^47^ were selectively detected by cross polarization (CP)^48^ using a 1 ms contact time.

**Fig. 3:**
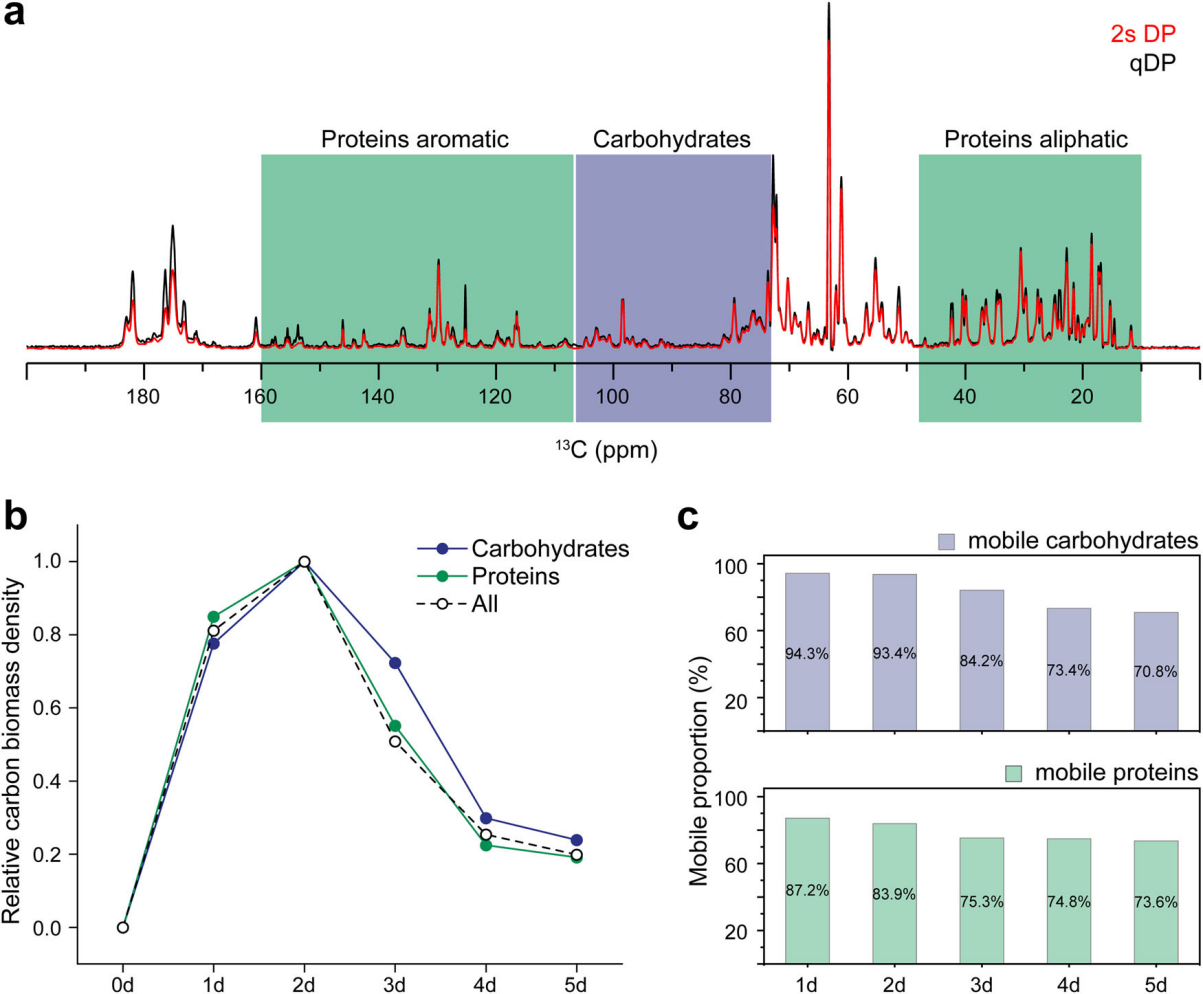
Evolution of carbohydrates and proteins within biofilm during biofilm development. **a** Overlay of 1D 2 s DP and quantitative DP (qDP) spectra. Regions used for quantification of carbohydrates and proteins are labeled and marked with different colors. **b** Relative carbon biomass density of carbohydrates and proteins during biofilm development. Each component is internally normalized using the Day-2 sample as the reference value of 1.0. **c** Mobile proportion of carbohydrates and proteins during biofilm development.

Quantitative analysis of 1D spectra provided time-resolved data on the temporal trends of total biomass density (Fig. 2b), carbohydrate and protein biomass densities (Fig. 3b), as well as the proportions of their mobile fractions (Figs. 2d and 3c). Quantification was performed using the integral. Data were normalized to account for the variations in the sample weights and number of scans (NS) for each spectrum. The proportion of mobile fraction was derived by comparing total or relevant region(s) in 2 s DP and quantitative DP spectra. Specifically, the spectral region of 74–105 ppm was used to quantify carbohydrates, while the combined ranges of 10–29 ppm, 34–48 ppm, 106–124 ppm, 132–160 ppm were assigned to proteins for quantification. Detailed data and calculations are provided in Supplementary Tables 2–5.

Two-dimensional (2D) NMR spectroscopy techniques were employed to enhance the resolution in detecting biofilm compositions within distinct dynamics regimes. The 2D ^13^C–^13^C DP refocused *J*-INADEQUATE (incredible natural abundance double quantum transfer experiment) spectra^49,50^ were acquired to resolve mobile components with through-bond connectivity. The 2D ^13^C–^13^C dipolar-assisted rotational resonance (DARR) spectra^51^ with 50 ms mixing time were collected to probe the rigid components within the biofilm samples. Most assignments are identical with those reported in our previous work^45^. A few newly emerged signals for carbohydrates and aliphatic carbons were observed and are listed in Supplementary Tables 6 and 7.

**Fig. 4:**
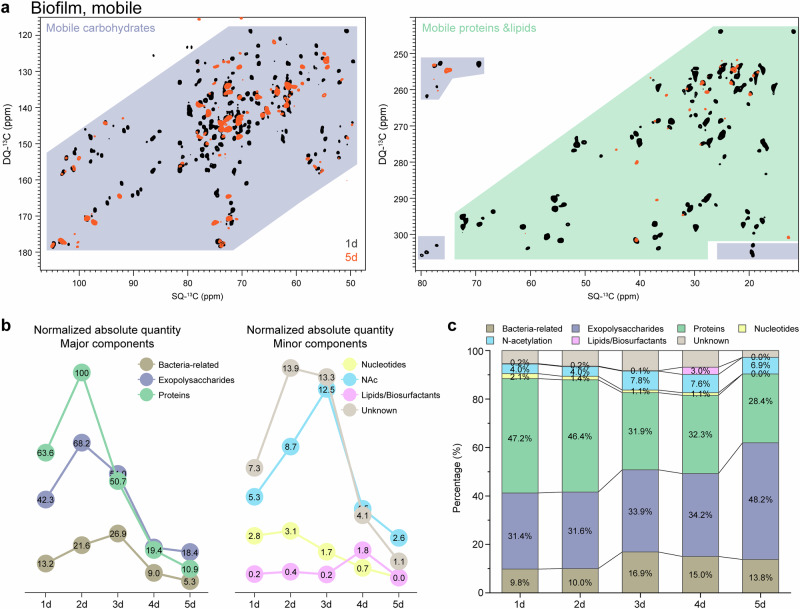
Compositional analysis of mobile phase within *B. subtilis* biofilm. **a** Overlay of 2D ^13^C–^13^C *J*-INADEQUATE spectra of biofilm on day 1 (black) and day 5 (red). Light blue and light green highlight the regions of mobile carbohydrates and proteins/lipids, respectively. The spectra are processed with identical parameter settings to ensure consistency. Twenty-four contour levels were plotted, starting from a minimum level of 15.3% of the G1 peak (170.9–98.8 ppm) in each spectrum, with a multiplication factor of 1.175. **b** Normalized absolute quantities and **c** relative proportions of components within the mobile phase during biofilm development (Supplementary Tables 8 and 9). In **b**, the protein quantity on day 2 is arbitrarily set as the reference point of 100.

**Fig. 5:**
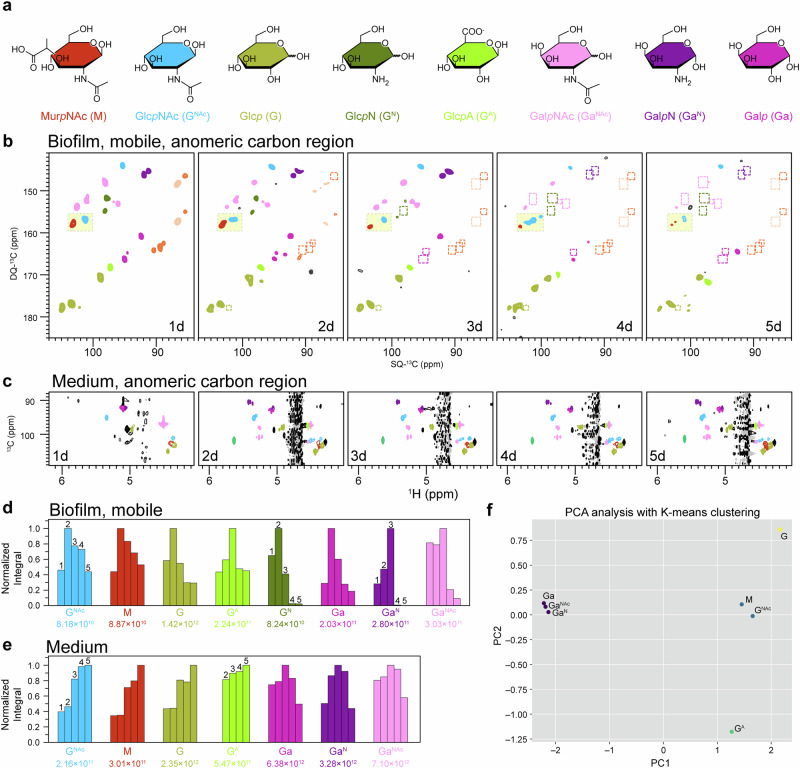
Compositional analysis of exopolysaccharides. **a** Representative chemical structures of sugar units detected within biofilm. Anomeric carbon region of the **b** 2D ^13^C–^13^C DP-based refocused *J*-INADEQUATE spectra and **c** 2D ^1^H–^13^C HSQC spectra. Signals from bacteria cell wall polymers and exopolysaccharides are color-coded as shown in panel **a**. In panel **b**, signals from bacteria cell wall polymers are highlighted with yellow boxes. Disappeared peaks are marked with dashed-line boxes. The INADEQUATE spectra were plotted using the parameters as described in Fig. 4. For the HSQC spectra, fourteen contour levels are plotted from a minimum level of 0.05% of the highest peak (23.4–1.80 ppm) of each spectrum with a multiplication factor of 1.5. Normalized quantities of monosaccharide compositions within **d** biofilm and **e** medium. Normalization is performed within each type, with the highest integral value labeled beneath. **f** Principal component analysis of monosaccharide compositions with K-means clustering.

Semi-quantification of the predominant mobile fraction in biofilm samples was conducted using 2D *J*-INADEQUATE spectra (Supplementary Table 8), following the same methodology as previously described^45^. The components are categorized to seven groups based on their distinct sources: bacteria-related, exopolysaccharides, proteins, nucleotides, N-acetyl group, lipids/biosurfactants and unknown. Their change trends in relative proportions and absolute quantities are calculated and summarized (Supplementary Table 9, Fig. 4b, c). The monosaccharide compositions of biofilm carbohydrates are grouped by their chemical type and then quantified to derive the temporal pattern in abundance (Supplementary Table 10, Fig. 5d).

The dynamics of exopolysaccharides were investigated by measuring ^13^C spin-lattice (T_1_) relaxation using the standard inversion recovery method. Relaxation data were fitted using the single-exponential decay function with Scipy^52^ (Supplementary Fig. 7, Supplementary Table 13). The dynamics of rigid phase, comprising bacteria cell wall polymers and protein fibril, were assessed using 1D ^13^C–^1^H dipolar-chemical-shift correlation (DIPSHIFT)^53^ experiments with CP for magnetization initiation. Frequency-switched Lee-Goldburg (FSLG)^54^ homonuclear decoupling was applied to the ^1^H channel with an effective field strength of 83.3 kHz. Dipolar dephasing curves were generated by plotting the intensities of well-resolved peaks against the dipolar evolution time, and were further simulated using the Python script^55,56^ downloaded from Dr. Mei Hong’s lab (https://meihonglab.com/hong-lab-software/) (Supplementary Figs. 8 and 9). The effective C–H coupling constants for CH or CH_2_ group and the order parameters are summarized in Supplementary Table 14.

### Simplified mathematical derivation of compositional proportions from consumption quantities

For the first order reaction, the degraded quantity is linearly proportional to the average concentration of the substrate as demonstrated by Eqs. (1)–(6).

1 $${Rate}=\frac{d\left[S\right]}{{dt}}=k\left[S\right]$$

2 $$\left[S\right]={C}_{0}{{\cdot }}{e}^{-{kt}}$$

$\left[S\right]$ is the substrate concentration, ${C}_{0}$ represents the initial concentration. The average concentration $\left\langle \left[S\right]\right\rangle$ can be calculated and simplified as following:

3 $$\left\langle \left[S\right]\right\rangle =\frac{1}{t}{\int }_{\!\!0}^{t}\left[S\right]{dt}$$

4 $$\left\langle \left[S\right]\right\rangle =\frac{1}{t}{\!\!\int }_{0}^{t}{C}_{0}{{\cdot }}{e}^{-{kt}}{dt}=\frac{{C}_{0}}{{kt}}\left(1-{e}^{-{kt}}\right)$$

The degraded quantity can be calculated as:

5 $$D=\left({C}_{0}-{\left[S\right]}_{t}\right){{\cdot }}V={C}_{0}\left(1-{e}^{-{kt}}\right){{\cdot }}V$$

$D$ is the degraded quantity, $V$ is the volume. Substituting Eq. (4) into Eq. (5) yields the linear correlation between the degraded quantity and the average concentration.

6 $$D=\left\langle \left[S\right]\right\rangle {{\cdot }}{kt}{{\cdot }}V$$

It has been measured that the degraded quantity on day 3 and day 4 are 49.28 and 31.31 for proteins, 14.18 and 33.43 for carbohydrates. We can easily derive their relative proportions within the degraded matrix using Eqs. (7)–(10), with the simplified assumption of equal volume and total biomass.

7 $$p1+c1=100 \%$$

8 $$p2+c2=100 \%$$

9 $$\frac{p1}{p2}=\frac{{\left[{Prot}\right]}_{1}}{{\left[{Prot}\right]}_{2}}=\frac{49.28}{31.31}$$

10 $$\frac{c1}{c2}=\frac{{\left[{Carbo}\right]}_{1}}{{\left[{Carbo}\right]}_{2}}=\,\frac{14.18}{33.43}$$

The variables $p1$, $p2$, $c1$, $c2$ represent the compositional percentages of proteins and carbohydrates on day 3 and day 4, respectively. By solving the Eqs. (7)–(10), these proportions are determined to be 79% ($p1$), 50% ($p2$), 21% ($c1$), and 50% ($c2$).

### Principal component analysis (PCA) of monosaccharide compositions

Monosaccharide compositions were analyzed over a period from day 2 to day 5. For each monosaccharide type, a ratio was calculated by dividing the integral value in the biofilm by that in the corresponding spent medium. This resulted in a vector of length 4 for each monosaccharide type, representing the time points from day 2 to day 5. The data was standardized using the ‘StandardScaler’ function provided in the Scikit-learn package^57^, which adjusts each feature to have a mean of 0 and a standard deviation of 1. This standardization ensures all features are equally weighted and allows for more accurate analysis. PCA was conducted in two dimensions. The first two principal components together accounted for approximately 98.5% of the variance in the data. K-means clustering was then applied to the PCA-transformed data to categorize the samples into four distinct groups. This choice was guided by quantitative analysis, including the Silhouette score^58^, as well as prior biological knowledge, such as the expectation that peptidoglycan components GlcNAc and MurNAc would form a distinct class. The raw data and processed data are summarized in Supplementary Table 12.

## Results

### Asynchronous digestion/degradation of mobile and rigid phases in biofilm

In this study, we utilized a modified MSgg medium^46^ to culture biofilm pellicles of the *B. subtilis* NCIB3610 strain for a total of 5 days. Samples were harvested every 24 h for analysis (Fig. 2a). This modified medium, compared to the conventional MSgg medium, doubles the concentrations of carbon and nitrogen sources (glycerol and glutamate) while significantly reducing the buffer MOPS concentration (100 mM to 5 mM). These modifications offer several advantages, including higher carbon isotope labeling efficiency and faster biofilm formation/dispersion, thereby facilitating accurate measurement of prominent variances within the experimental time window by ssNMR.

It is essential to recognize that the samples collected over time are inherently from different wells, which prevents direct comparison of absolute quantities due to the lack of a common reference base. Instead, calibrated metrics, such as weight percentages, provide a more consistent basis for comparison across different samples. For instance, the total integral of a spectrum is directly proportional to the absolute carbon mass of a sample. Calibrating this integral with both sample weight and the number of scans (NS) performed during data collection yields a normalized value, which correlates linearly with the carbon biomass weight percentage in the sample. For simplicity, we define this calibrated quantity as ‘carbon biomass density’. In the following text, the analysis of fluctuations in the quantities of different groups or components over time will refer exclusively to calibrated values, specifically carbon biomass density.

The analysis of the temporal dynamics of carbon biomass density, quantified by 1D quantitative spectroscopy (Fig. 2b, Supplementary Table 2), reveals a rapid initial accumulation phase during which the biofilm acquires the majority of its biomass within the first 24 h. The biofilm then reaches its peak biomass on day 2, followed by a marked decline over the subsequent 48 h. This decline continues at a reduced rate, with the biomass ultimately decreasing to 20% of its maximum value on day 5. Analysis of the spent medium reveals that the carbon source, glycerol, is depleted within 48 h (Fig. 2c, Supplementary Table 1), coinciding with the peak biomass in the biofilm. This depletion triggers the onset of the dispersal process, leading to the observed biomass loss in the following days.

Our previous work has revealed that while over 90% of the carbon biomass in mature biofilm are in mobile state, a small but notable portion exhibits substantial rigidity^45^. To assess changes in the distribution of domains with varying mobility over time, we quantified the mobile phase, using 1D DP spectra with a 2 s recycle delay, a time interval suitable for selectively detecting the mobile compositions with fast ^13^C-T_1_ relaxation. Our data reveal a stable 89–91% mobile phase during the maturation stage (Fig. 2d, Supplementary Table 3). As the dispersal process initiates on day 3, the mobile phase content plunges to 77%, and remains steady at ~76% in the next 2 days. While the rigid phase is not directly quantifiable, it can be loosely estimated by the difference between the total and mobile phases. Overall, the observed decrease in the relative proportion of the mobile phase (91% to 76%), along with an increase in the combined proportion of the rigid phase (~9% to ~24%), suggests that the mobile phase is more susceptible to degradation compared to the rigid phase.

### Distinct behaviors in phase distribution and degradation of proteins and carbohydrates in biofilm

As the *B. subtilis* biofilm is predominantly composed of proteins and carbohydrates^45,59^, we further examine the biomass variations of the two major compositions, proteins and carbohydrates, by quantifying their distinct and non-overlapping regions in the 1D spectra. Specifically, 74–105 ppm is integrated to assess carbohydrates, while the 10–48 ppm and 106–160 ppm ranges, excluding lipid signals, are used to evaluate proteins (Fig. 3a). Although ignoring the overlapped regions leads to underestimations of the total content, the specific partial biomass still provides a consistent approximation for tracking the fluctuations of the component’s total biomass.

The temporal biomass profile of proteins more closely mirrors that of total biomass compared to that of carbohydrates (Fig. 3b, Supplementary Table 4). This observation is consistent with the higher proportion of proteins (~50%) relative to carbohydrates (~30%) in the biofilm makeup of *B. subtilis*^45^. The greater abundance of protein naturally leads to its stronger correlation with the biofilm total biomass in the overall trend of absolute quantity.

The mobility distributions of carbohydrates and proteins exhibit distinct patterns (Fig. 3c, Supplementary Table 5). Throughout the observation period, the fraction of mobile carbohydrates fluctuates significantly, while the proportion of mobile proteins is comparatively less varied. For carbohydrates, the mobile phase starts with a dominant high percentage of approximate 94% in the first two days. This proportion rapidly decreases to 84% on day 3 and further to 71% on day 5. Given that the mobile phase of carbohydrates is dominated by exopolysaccharides, while the rigid carbohydrates are exclusively derived from cell wall peptidoglycans^45^, the observed variations in their proportions primarily reflect the distinct regulatory dynamics between matrix exopolysaccharides and rigid cell clusters throughout the biofilm life cycle. For proteins, the mobile fraction shows less variability compared to carbohydrates, gradually decreasing from an initial 87% to a final 74%.

Based on the observed biomass variation patterns, we tentatively divide the 5-day period into three stages: the maturation stage (days 1–2), during which the biofilm grows and accumulates continuously. The mobile phase maintains at a high, stable percentage of ~90% of total biomass. Meanwhile, the major components exhibit a consistent partitioning proportion into the mobile phase, with mobile carbohydrates and proteins accounting for 94% and 87% of their respective total biomasses. The observed consistency in phase distribution suggests a finely tuned balance in the biofilm’s structural organization in the formation stage, characterized by abundant nutrients and favorable environmental conditions; the fast decay stage (days 3–4), marked by rapid biomass reduction in both proteins and carbohydrates. During this period, mobile carbohydrates and proteins undergo substantial degradation and release. As a result, their relative proportions plummets from 94% to 84% and 87% to 75%, respectively; the slow decay stage (day 5), when the rate of biomass loss notably slows down following the dispersal of the majority of the biomass. Concomitantly, the mobile proportions of the primary components also reach a more stable state, exhibiting only a slight decline. This stage represents a late-phase scenario where the residual biomass exhibits increased recalcitrance, persisting in the degraded environment.

### Nuanced trends in biofilm dispersion revealed by detailed quantitative analysis of the mobile phase

The mobile phase comprises 76–91% of total biomass throughout the experiment (Fig. 2d), making its detailed compositional analysis crucial for understanding biofilm dynamics across development and dispersion stages.

High-resolution compositional quantification was achieved using 2D refocused *J*-INADEQUATE spectroscopy with a 2 s recycle delay (Fig. 4a, Supplementary Table 8). Detected signals are mostly classified into three main categories based on their definitive sources: proteins, exopolysaccharides and cells (including peptidoglycans, wall/lipid teichoic acids (WTA/LTA)). Minor signals from nucleotides and lipids/biosurfactants (aliphatic chains) are listed separately. Signals from chemical groups with ambiguous sources, such as N-acetyl groups, are categorized independently. Any unassigned signals are grouped as unknown.

Variations in the absolute quantities of different components over time reveal distinct patterns. While both proteins and exopolysaccharides peak on day 2 before declining, their dispersal is asynchronous, with the steepest decreases occur on day 3 for proteins and day 4 for exopolysaccharides (Fig. 4b, Supplementary Table 9). By day 5, both of their decline rates slow down considerably. The observation, potentially attributable to a multitude of factors, is more plausibly explained by the heterogeneous distribution of proteins and exopolysaccharides. In a simplified model where the degradation rate solely depends on the local substrate concentration and the overall carbon density remains invariant, the degraded matrix comprises 79% proteins and 21% carbohydrates on day 3, and an equal proportion of proteins and carbohydrates on day 4 (see details in Methods section). The overall dispersion process results in a reversal of the relative proportions of proteins and carbohydrates from day 1 to day 5, Specifically, percentages of proteins and carbohydrates shift from 47.2% to 28.4% and from 31.4% to 48.2%, respectively (Fig. 4c, Supplementary Table 9). Besides the two major components of proteins and exopolysaccharides, minor nucleotides and the unknown group also reach maximum level on day 2. By day 5, the nucleotides fall below the detection limit.

Comparatively, cell-surface anchored peptidoglycans and teichoic acids, along with the N-acetylation (NAc) group, exhibit a delayed mass accumulation, culminating on day 3 (Fig. 4b, c). It’s important to note that the quantity of cell surface carbohydrates serves as a complex indicator. While it generally correlates with cell numbers, these carbohydrates also increase in response to various stress. The observed moderate increase from 21.6 to 26.9 within 48–72 h, a time window with the most dramatic decay of extracellular matrix, likely reflects an adaptive overproduction triggered by nutrient depletion rather than a proportional increase in cell count. Likewise, the peaking lag of the NAc group may also be indicative of an adaptative response as N-acetylation of proteins and lipids are known to be directly or indirectly involved as part of stress response^46,60–62^. The two groups exhibit closely correlated trends in their relative proportions, hinting at a potential synchronized responsiveness.

Another striking change is observed in the signals from aliphatic chains, which surge remarkably on day 4 (Supplementary Fig. 5). The absolute quantity increases by nearly an order of magnitude from the previous day, rising from 0.2 to 1.8 (Fig. 4b). Considering the drastic loss of biomass, the corresponding relative proportion jumps 30-fold, from 0.1% to 3.0% (Fig. 4c). While aliphatic carbons are often sourced from lipids, in our specific context, the quantity spike is more likely due to certain secreted biosurfactants, which facilitate cell dispersal in the late stage^15,63,64^.

It is worth noting that although the rigid phase represents a minor fraction and is not directly quantifiable due to the non-quantitative nature of the CP-based spectra, qualitative evaluation still provides valuable insights into its changes throughout the life cycle. The rigid phase primarily consists of aggregated cells and TasA fibers^45^. A comparison of the spectra from day 1 and day 5 reveals significant weakening of signals for both components (Supplementary Fig. 4), with TasA fibers showing a more pronounced reduction. Additionally, notable peak shifts in protein regions suggest that the dispersal process might involve considerable structural rearrangements for the rigid protein fibers. Another notable observation is that the apparent signal intensities of cells continuously strengthen until day 2. While the signal enhancement in CP spectra could be attributed to both an increase in quantity and improved structural rigidity, the observed change, regardless of contributing factors, indicates a structural reinforcement within the cells. It is tempting to associate the robust cell aggregates, exhibiting rigidity comparable to that of wood cellulose^45^, with the most persistent subpopulation of cells in the biofilm. The key roles these cells play in biofilm resistance, in conjunction with their delayed development, align with the well-established fact that premature biofilms are more susceptible to disruption compared to mature biofilms^65–67^.

### Differential partitioning of galactose-based and glucose-based exopolysaccharides in biofilms and medium

Given the structural and compositional complexities, characterization of carbohydrate compositions typically relies on analyzing the profiles of the constituting monosaccharides. Temporal fluctuations of these sugar units in both biofilm and spent medium can provide insights into the metabolism of structural exopolysaccharides within the biofilm and its exchanges with the environment.

Quantitative analysis is conducted using 2D DP *J*-INADEQUATE spectra for the biofilm mass and HSQC spectra for the spent medium, respectively. Consistent with our previous work^45^, eight types of sugar units are identified in the biofilm samples (Fig. 5a, Supplementary Table 6). Apart from N-acetylmuramic acid (MurNAc), these sugars are based on either glucose (Glc) or galactose (Gal). All these sugar units, except glucosamine (GlcN), are also detected in the spent medium (Fig. 5b, c, Supplementary Fig. 6). These monosaccharides, excluding N-acetylglucosamine (GlcNAc) and MurNAc, constitute the major composition of exopolysaccharides in the biofilm matrix. However, the exact structural arrangements connecting these units is largely unknown yet. Through analyzing their metabolic variations, we aimed to search for grouped behaviors that could provide insights into their organizational patterns.

Using internal calibration, we analyzed the quantity change profiles of each sugar unit over the 5-day period in both the biofilm samples and the spent media (Fig. 5d, e, Supplementary Table 10). While most sugar units in the biofilm samples peak on day 2, paralleling the overall biomass. glucuronic acid (GlcA), galactosamine (GalN) and N-acetylgalactosamine (GalNAc) reach their maximum levels on day 3. This delayed accumulation, indicative of delayed biosynthesis and/or delayed degradation, suggests that these sugars might be more involved in stress or adaptative responses rather than the primary metabolism. Notably, both GlcN and GalN are rapidly exhausted after day 3. Comparatively, Glc-based sugars retain more biomass (30–45%) than Gal-based sugars (<20%) in the end. In the spent medium (Fig. 5e, Supplementary Table 11), the overall trends for changes in sugar unit quantities are clearly divided into two groups: one group, including all types except Gal-based sugars, shows continuous growth throughout the period, while the other group, consisting of Gal-based sugars, shows a decline after day 3.

Analysis of absolute quantities reveals more striking differences: Glc is the predominant sugar type in biofilm carbohydrates, with normalized biomass 4–10 times higher than the average of other sugars throughout the experimental timeframe (Fig. 5d). In contrast, Gal-based sugars are far more abundant in the medium, exhibiting 2–8 times higher concentrations compared to the average of other sugars (Fig. 5e). This observation further underscores the distinct metabolic dynamics of Glc and Gal sugars.

We further conducted a principal component analysis (PCA)^68^ on the dynamic changes of the sugar units (Fig. 5f, Supplementary Table 12). To incorporate the differences in absolute quantities between the biofilm and medium, we calculated the ratio of absolute sugar quantities in the biofilm to those in the spent medium. To capture the temporal trends, data from days 2 through 5 were included, as the GlcA data was missing from the spent medium on day 1. PCA analysis reveals that Gal-based sugars are in very close proximity; GlcNAc and MurNAc are relatively close; In contrast, Glc and GlcA are far apart from each other and from other sugar units as well. Application of K-means clustering algorithms yields an optimal classification of four distinct clusters: (1) Gal-based sugars, (2) GlcNAc and MurNAc, (3) Glc, and (4) GlcA. The pronounced clustering of Gal-based sugars strongly indicates that they form a distinct exopolysaccharide class, exhibiting comparable metabolic behaviors within the biofilm matrix and analogous partitioning between the biofilm and surrounding medium. GlcNAc naturally clusters with MurNAc as both are primary monosaccharides of cell wall peptidoglycans. Glc, with its predominant presence in exopolysaccharides, conceivably forms the prevalent structural glucans. GlcA exclusively exists as side chains and likely derivatizes the glucans. The distinct patterns between Glc and GlcA suggest pronounced differences in their metabolic dynamics. This is evident from the delayed accumulation of GlcA and its notably higher retention percentage compared to Glc in the biofilm samples (Fig. 5d). Specifically, the higher retention of GlcA is likely due to its ability to chelate Ca^2+^ ions from the medium. This chelated structure is well-known to strengthen the biofilm matrix and is intrinsically more resistant to enzymatic degradation^42,69,70^.

### Mobility variation of mobile and rigid phase

Beyond compositional variances, molecular dynamics provide another crucial measure for assessing the evolution of biofilm structure. Our recent study has revealed that exopolysaccharides exhibit narrow range of relaxation times, indicative well-formed structural framework^45^. Therefore, in the dominant mobile phase, investigation focus is placed on the carbohydrate components. The minor yet crucial rigid phase is as well examined to provide further insights into its structural and functional significances.

**Fig. 6:**
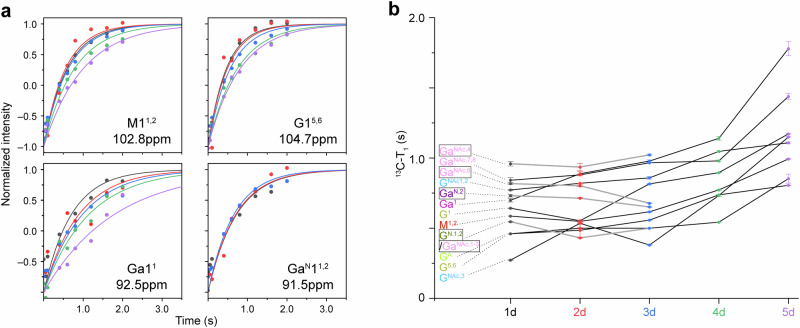
Changes in ^13^C-T_1_ relaxation times of exopolysaccharides during biofilm development. **a** Representative ^13^C-T_1_ relaxation simulation curves of monosaccharide units in exopolysaccharides. **b** Temporal profiles of ^13^C-T_1_ relaxation time constants from various sugar units. The subtypes absent after day 3 are highlighted with boxed labels.

In the mobile phase, the mobility of carbohydrates is assessed by measuring the relaxation times of the C1 carbon atoms in the monosaccharide units (Fig. 6, Supplementary Fig. 7, Supplementary Table 13). For the first 3 days, the relaxation times for various sugar units are relatively stable with minimal changes. A notable increase begins on day 4 and becomes more pronounced by day 5. Increased relaxation times generally indicate reduced flexibility, reflecting more restricted molecular or structural movement. This observation aligns with the general assumption that, flexible domains are more prone to degradation compared to their more rigid counterparts, which gain rigidity through tight intra- and inter-molecular packing or chelation with metal ions. The notable increase in relaxation times occurs after nearly 70% of exopolysaccharides have been degraded, suggesting these relatively rigid regions comprise a minor fraction of the exopolysaccharide framework.

**Fig. 7:**
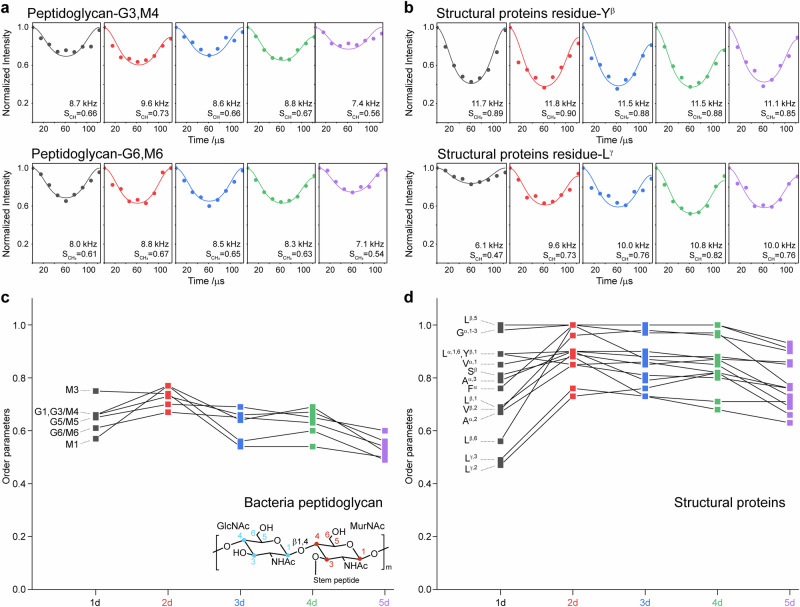
Steady molecular dynamics of rigid phase during biofilm development. Representative dipolar dephasing curves of **a** bacteria peptidoglycan and **b** proteins. Dots indicate the data points extracted from DIPSHIFT experiments, while lines represent the best-fit simulations. The assignments, best-fit dipolar couplings, and order parameters are labeled. Steady trends in rigidity for **c** bacteria peptidoglycan and **d** proteins from day 1 to day 5.

Dynamic properties of the minor rigid phases are evaluated using DIPSHIFT experiments. The derived order parameter, ranging from 0 to 1, serves as the metric for rigidity, with higher values indicating greater rigidity. The rigid phase is predominantly composed of cells and structural proteins, with a trivial amount of lipid vesicles^45^. While the protein components, mainly TasA fibrils, provide some mechanical support to the cell aggregates, they primarily form their own structural domains with minimal interweaving^45^. Mobility analysis of representative carbon sites within cell wall peptidoglycans and protein amino acids indicates that both domains maintain relatively consistent rigidity throughout the experimental period (Fig. 7, Supplementary Figs. 8 and 9, Supplementary Table 14). Cell aggregates show a slight increase in rigidity from 0.57–0.75 on day 1 to 0.67–0.77 on day 2, followed by a gradual decrease to 0.49–0.60 by day 5. For proteins, the collective rigidity also increases from 0.47–1.00 on day 1 to 0.73–1.00 on day 2. The subsequent decrease is minimal, with rigidity remaining at 0.63–0.93 on day 5. These consistency in molecular dynamics with degradation suggests, unlike exopolysaccharides in the mobile phase that form a macrostructural network, the rigid phase consists of numerous isolated, resilient seeds. These seeds largely maintain their structural integrity and exhibit stable dynamic features throughout the ongoing degradation process.

## Discussion

**Fig. 8:**
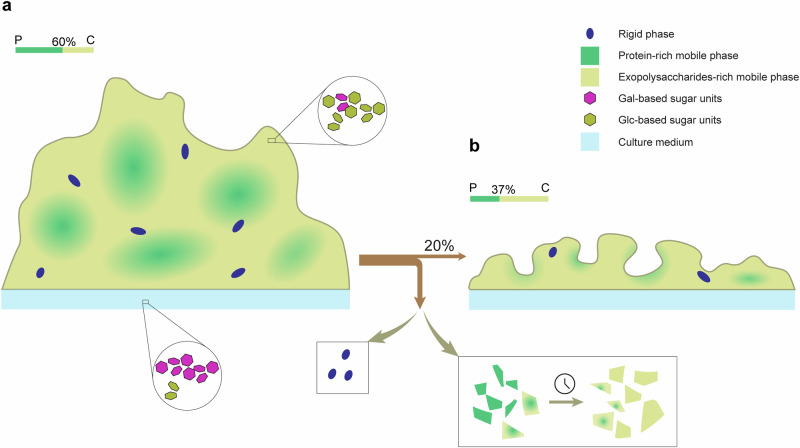
Conceptual model of *B. subtilis* biofilm dispersal. *B. subtilis* biofilm at (**a**) maturation stage (day 2) and (**b**) late dispersal stage (day 5). Biofilm structure is illustrated with distinct dynamics regimes: the rigid phase (blue, oval dots) and the mobile phase, comprising protein-rich regions (green) and exopolysaccharide-rich regions (yellow). Overall, the carbon biomass density decreases by 80% after dispersal. In the predominant mobile phase, significant proportional changes in proteins and polysaccharides (stacked bar in the top-left corner) suggest distinct spatial distributions. Degradation starts in protein-rich regions and gradually extends to exopolysaccharide-rich regions (rectangular box), indicating that proteins are spatially closer to the cells, which secret the disrupting enzymes, compared to exopolysaccharides. The rigid seeds are likely to be released with minimal structural damage (cubic box). The monosaccharide compositions of exopolysaccharides differ significantly between the biofilm and the spent medium (zoomed-in circles): the biofilm is rich in glucose-based sugars (olive), while the spent medium is abundant in galactose-based sugars (magenta). This trend is consistent throughout the biofilm’s life cycle.

Leveraging the unique capabilities of ssNMR spectroscopy, our study offers a comprehensive examination of *B. subtilis* biofilm development over a 5-day period. Through the non-destructive, quantitative assessment of structural components in situ and the unique analysis of distinct dynamic regimes within the biofilm, we delineate the detailed compositional and structural variations across the formation and dispersal stages. These time-resolved changes greatly enhance our understanding of the precise temporal dynamics of matrix degradation during biofilm dispersion. They offer crucial insights (Fig. 8) into the regulatory mechanisms that orchestrate the metabolism of key structural components, and are of great practical importance for optimizing biofilm intervention strategies in both medical and industrial applications.

Both mobile phase and rigid phase coexist throughout biofilm development process. While their proportions remain relatively stable at 24 and 48 h, significant variations emerge once dispersal stage begins, suggesting differential metabolic patterns for these structurally distinct domains (Figs. 2b, d and 3c). The apparent proportional stability observed between 24 and 48 h, coupled with similar sample morphology and comparable overall biomass, indicates the selected time points fall within the metabolically steady maturation and maintenance stage. Potential notable differences likely exist within the first 24 h of the initial formation stage for these phases, which were overlooked in this study and require more detailed investigation specifically during the initial formation stage. In addition to the marked proportional variation, another striking difference is the distinct trends of molecular dynamics between the two phases during the dispersal stage (Figs. 6 and 7). Compared to the notably increased rigidity in the mobile phase, the rigidity of the rigid phase exhibits minimal change. These findings suggest that the predominant mobile phase behaves as a dynamically heterogeneous and macro-scale matrix, which gradually becomes more rigid as degradation preferentially targets its more flexible and exposed regions. In contrast, the minor rigid domain likely exists as numerous independent micro-scale rigid seeds dispersed within the continuous mobile phase. During dispersal, these seeds are released individually with structural integrity largely maintained, thereby ensuring the observed consistent rigidity of the minor rigid phase throughout the process. It is important to note that although the rigid phase behaves like stable, discrete microdomains within the matrix, it remains unclear whether the contained cell aggregates represent specific cell types or cells in distinct metabolic or functional states. This ambiguity also extends to their behavior upon release. While studies have shown that dispersed cells exhibit enhanced mobility and virulence^7,71–73^, primarily due to gene expression regulation in response to environmental stress, it is unclear whether these released rigid seeds follow the similar pattern. One interesting functional hypothesis is that that these entities represent a specialized subpopulation characterized by exceptional structural integrity, potentially serving as highly effective nucleation points for new biofilm formation. Nonetheless, considerable future research is needed to elucidate the precise nature, behavior, and functional significance of these minor rigid domains, including their transcriptional profiles, metabolic states, and potential roles in biofilm life cycle.

The dissolution of the dense extracellular matrix is essential in dispersal process to facilitate the release of the encaged cells. Studies have demonstrated that a diverse array of carbohydrate-degrading and proteolytic enzymes can effectively disrupt early-stage biofilms^65–67^; however, their efficacy notably reduces when applied to mature biofilms, suggesting an increased hindrance in diffusion and penetration within the denser and more complex extracellular matrix. In our study, the dissolution enzymes are secreted by deeply embedded cells. Unlike externally added enzymes that must diffuse into the matrix, these endogenously produced enzymes face the opposite challenge: they must diffuse outward to facilitate matrix breakdown and eventual cell release. Consequently, the degradation profiles of the matrix components effectively capture the localized concentration gradients of these structural elements radiating from the deeply-embedded cells. Our data reveals that the dramatic degradation of proteins precedes the matrix exopolysaccharides by one day (Figs. 3b and 4b, c), suggesting proteins are probably more concentrated in the proximity of cells, exopolysaccharides are distributed farther from the cells. This observation is consistent with previous findings that employed confocal microscopy to examine the spatial architecture of biofilm components. One study revealed that proteins are concentrated around and within cell clusters in *Vibrio cholerae* biofilms^74^. Another study showed that in *Pseudomonas aeruginosa* biofilms, the exopolysaccharide Psl primarily accumulates around the edges of microcolonies, leaving the central regions largely devoid of it^75^. Our data and these studies highlight a shared structural pattern in biofilms (Fig. 8): cells tend to associate with proteins to form a protein-rich phase, while exopolysaccharides fill the interspaces, creating an exopolysaccharide-rich phase, which constitutes the overarching structural scaffold of the biofilm. This delineation underscores distinct functional roles within the biofilm, with proteins contributing to cellular cohesion and exopolysaccharides providing structural support to the overall matrix. Furthermore, the differentiated breakdown of proteins and exopolysaccharides observed in our study, remains mechanistically obscure. Further investigation is necessary to determine whether this process is passively controlled by intrinsic concentration gradients or more precisely regulated at the gene expression level by cells responding to environmental changes.

Another drastic sequential event during dispersal is the spike in secretion of components rich in aliphatic carbons (Fig. 4b, c, Supplementary Fig. 5), also occurring after dramatic breakdown of proteins. Although the precise molecular identities of these components could not be confirmed, the timing of their production during the dispersal stage strongly suggests that they are likely the cell-secreted biosurfactants, which play crucial functional roles in the detachment and release of biofilm cells. Moreover, the precise timing of secretion after significant dissolution of proteins suggests it is a tightly regulated process. When the local cellular environment becomes more diluted and permeable, it facilitates the diffusion of these biosurfactant molecules and greatly enhancing their efficacy. Overall, these temporal profiles of different components are intricately linked to the structural architecture of the biofilms, which actively influences various processes throughout the biofilm lifecycle.

Previous studies have revealed that in *B. subtilis* biofilms, the matrix exopolysaccharides are primarily composed of Glc- and Gal-based monosaccharides^45,76^, yet their chemical structures remain undefined. Our study extends this understanding by examining the temporal patterns of these constituting sugars during biofilm development. We have uncovered that Glc-based polysaccharides exhibit a greater tendency to be retained within the biofilm structure, whereas Gal-based polysaccharides are more readily released into the spent medium (Fig. 5b–e). This differential behavior suggests distinct structural and functional roles for these two polysaccharide types within the biofilm matrix. We hypothesize that the greater retention of Glc-type exopolysaccharides implies that they likely have longer chains, are more insoluble, or they may have stronger/more interactions with another matrix component. Functionally, they serve as the primary structural scaffold of the biofilm, providing mechanical stability and cohesion to the overall matrix. In contrast, the Gal-type exopolysaccharides are more soluble and likely shorter-chain. The ease of diffusion and release suggest they may be more involved in various signaling process, or enable biofilm to rapidly restructure in response to environmental cues. Interestingly, recent research has highlighted the pivotal role of galactose metabolism in biofilm formation of *B. subtilis*^76^. While *B. subtilis* can utilize and convert environmental galactan into galactose and UDP-Gal, the intracellular accumulation of UDP-Gal is toxic. This toxicity is mitigated during biofilm formation by the production of EPS galactans. Thereby, the efficient release of EPS galactans into the surrounding medium provide a protective mechanism for the embedded cells from the toxicity induced by excessive galactans within the biofilm matrix. Our data reveals the sophisticated adaptation *B. subtilis* has evolved to delicately balance the benefits and potential toxicity of the ubiquitous carbon source, utilizing not only metabolic mechanism, but also the physical and structural aspects of biofilm and its components.

In conclusion, our work provides a comprehensive roadmap of *B. subtilis* biofilm development process, reveals novel insights into the structural and functional mechanisms underlying the adaptation and resilience of the biofilm matrix, and pave the way for future research exploring the effects of various strategies interfering biofilm development in the industrial and medical settings.

## Supplementary information

editorial-policy-checklist-rev1

reporting-summary-rev1

SI-dispersion-Rev1_unmarked

## Data Availability

All NMR spectra and analysis in this study are provided in the article and Supplementary Information. NMR raw data in this study is available upon request to the authors.
