# Supplementary material for: Time-resolved compositional and dynamics analysis of biofilm maturation and dispersal via solid-state NMR spectroscopy
Source: NPJ Biofilms Microbiomes. 2025 Jan 29;11:21. doi: 10.1038/s41522-025-00655-4 (PMC11779841; doi:10.1038/s41522-025-00655-4)
Supplement: Supplementary file 3 — SI-dispersion-Rev1_unmarked [file 41522_2025_655_MOESM3_ESM.pdf]

Time-resolved compositional and dynamics analysis of biofilm maturation and dispersal via solid-state NMR spectroscopy

Yi Xue[1], Xue Kang[1]*

[1] Institute of Drug Discovery Technology, Ningbo University, Ningbo, Zhejiang, 315211, China

* To whom correspondence should be addressed

Xue Kang (Email: kangxue@nbu.edu.cn)

Table of Contents

Supplementary Fig. 1. 1D spectral reproducibility of *B. subtilis* biofilm samples··········3
Supplementary Fig. 2. Analysis of the washing supernatant in sample preparation··········4
Supplementary Fig. 3. Stability of *B. subtilis* biofilm samples··········5
Supplementary Fig. 4. Temporal changes in the rigid phase of *B. subtilis* biofilm··········6
Supplementary Fig. 5. Evolution of lipids/biosurfactant signals in the mobile phase··········7
Supplementary Fig. 6. Representative anomeric carbon signals of monosaccharide··········8
Supplementary Fig. 7. Selective $^{13}C\text{-}T_1$ relaxation curves of exopolysaccharides··········9
Supplementary Fig. 8. DIPSHIFT curves of bacteria cell wall polymers in the rigid phase··········10
Supplementary Fig. 9. DIPSHIFT curves of proteins in the rigid phase··········11
Supplementary Table 1. Glycerol consumption in the spent medium over the 5-day period··········12
Supplementary Table 2. Data analysis of relative carbon biomass density··········13
Supplementary Table 3. Data analysis of mobile phase proportion··········14
Supplementary Table 4. The temporal profiles of carbohydrates and proteins biomass density··········15
Supplementary Table 5. Calculation of mobile proportions of carbohydrates and proteins··········16
Supplementary Table 6. Chemical shifts of anomeric carbon and proton of exopolysaccharides··17
Supplementary Table 7. Chemical shifts of aliphatic carbons emerged on day 4··········18
Supplementary Table 8. Semi-quantification of the mobile molecule phase··········19
Supplementary Table 9. Summary of semi-quantitative analysis··········21
Supplementary Table 10. Monosaccharide compositions within biofilm··········22
Supplementary Table 11. Monosaccharide compositions within medium··········23
Supplementary Table 12. Raw data and calibrated data in principle component analysis··········24
Supplementary Table 13. $^{13}C\text{-}T_1$ relaxation time constants of carbohydrates··········25
Supplementary Table 14. Dipolar order parameter of rigid compositions··········26
Supplementary Table 15. NMR experimental details in this work··········27
Supplementary References··········28

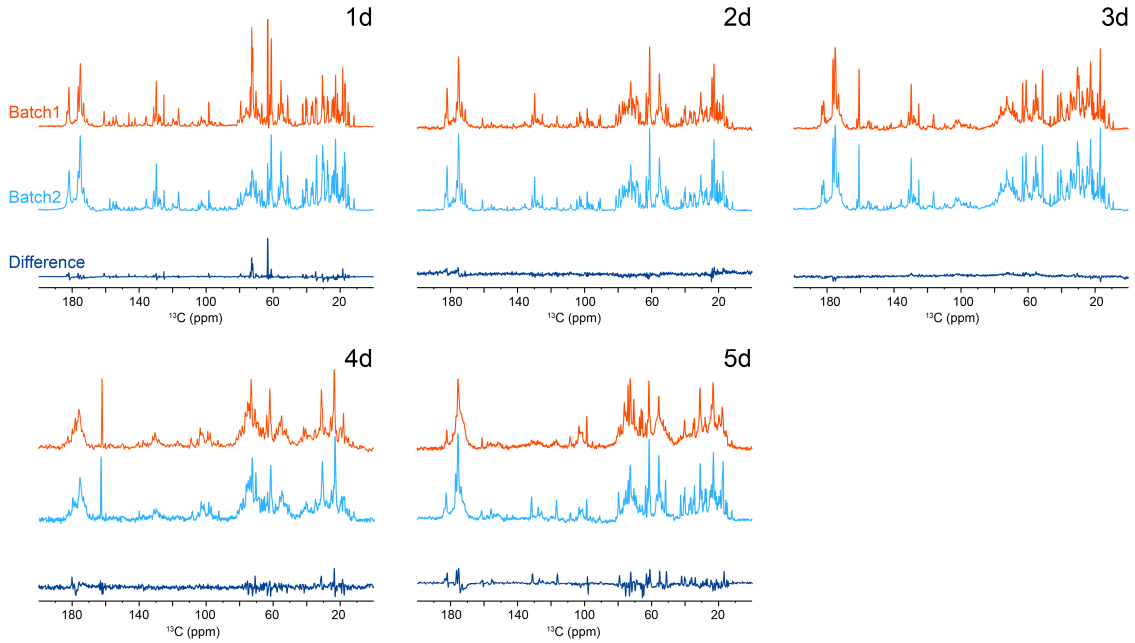

Supplementary Figure 1. 1D spectral reproducibility of *B. subtilis* biofilm samples. Quantitative 1D ^{13}C DP spectra (with a recycle delay of 15 s) of batch 1 (orange) and batch 2 (cyan) samples from day 1 to day 5. Difference spectra (blue) are calculated to highlight variations in peak profiles. No substantial peaks appear in the difference spectra, suggesting that both the composition and quantity of the samples demonstrate high reproducibility across the two separate batches. In the Day 1 replicates, the narrow peaks at 73 ppm and 63 ppm likely originate from glycerol molecules adhering to biofilms. The relatively noisier regions observed on Days 4 and 5 are probably due to the significantly lower biomass, resulting in increased noise. While both batch samples were analyzed with 1D ssNMR spectra, only batch 2 was used in the comprehensive 2D ssNMR analysis.

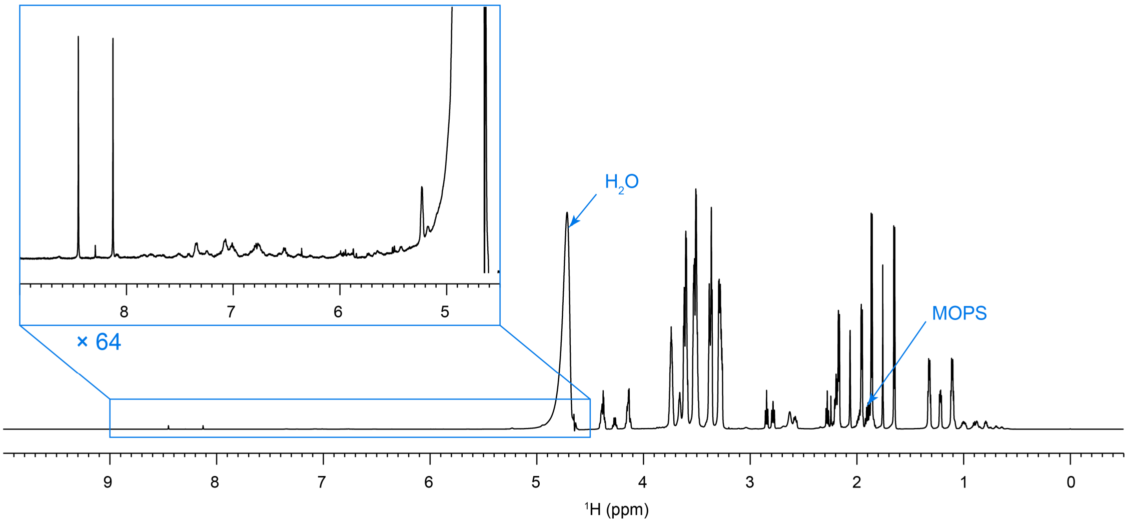

Supplementary Figure 2. Analysis of the washing supernatant in sample preparation using solution-state NMR. The 1D ^{1}H spectrum of the washing solution shows signals primarily between 1–5 ppm, indicating that small molecules are the main contributors. Signals of water and 3-morpholinopropane-1-sulfonic acid (MOPS, 5 mM in medium) are specifically labeled. A magnified view (64-fold) of the 4.5–9.0 ppm region highlights minimal detection of proteins and carbohydrates in the washing solution.

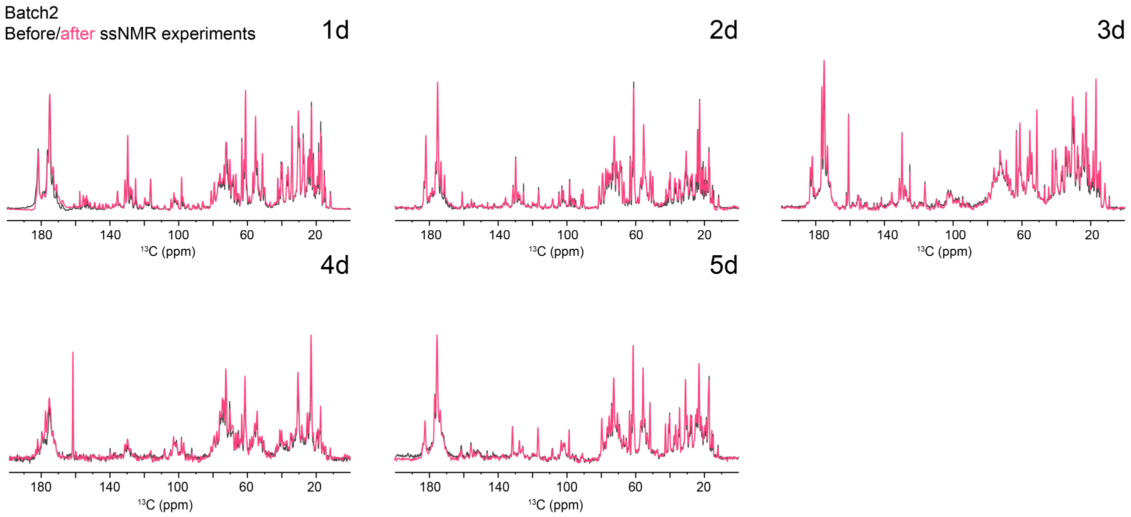

Supplementary Figure 3. Stability of *B. subtilis* biofilm samples. Quantitative 1D ^{13}C DP spectra (with a recycle delay of 15 s) were collected to evaluate the stability of batch 2 biofilm sample before (black) and after (rose) the ssNMR experiments. No significant decomposition/degradation occurred during the experiments.

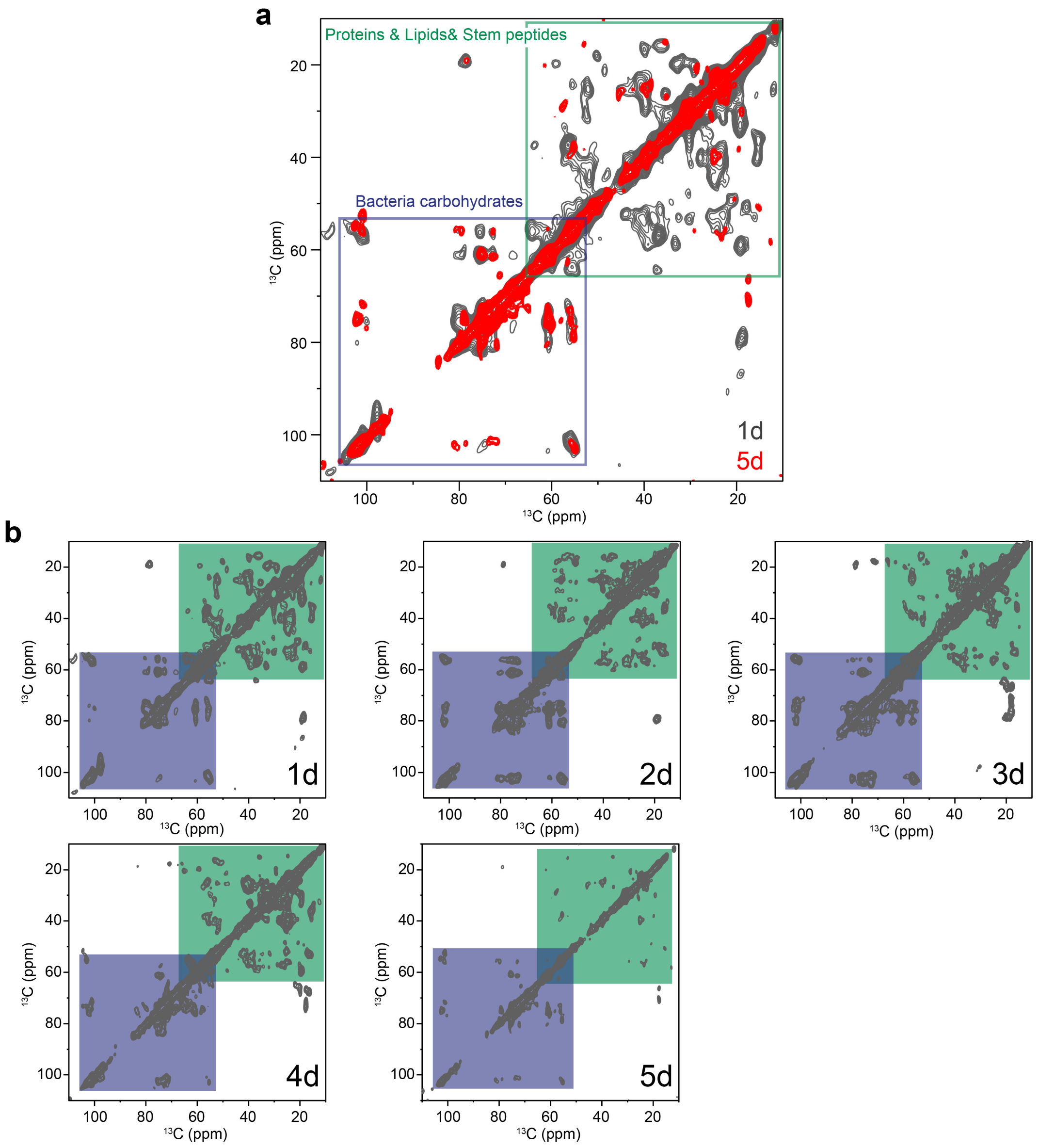

Supplementary Figure 4. Temporal changes in the rigid phase of *B. subtilis* biofilm. 2D ^{13}C–^{13}C correlation spectra of biofilm samples were measured using CP and 50-ms DARR mixing time for detecting TasA fibril and bacteria cell wall carbohydrates in the rigid phase. Regions corresponding to bacterial carbohydrates and proteins/lipids are highlighted with blue and green rectangles, respectively. (a) Overlay of spectra at Day1 (black) and Day5 (red). (b) Each spectrum from Day1 to Day5. To facilitate comparison, spectra in each panel are processed with same parameter settings. Thirty contour levels are plotted from a minimum level of 0.58% of the highest peak of each spectrum with a multiplication factor of 1.185.

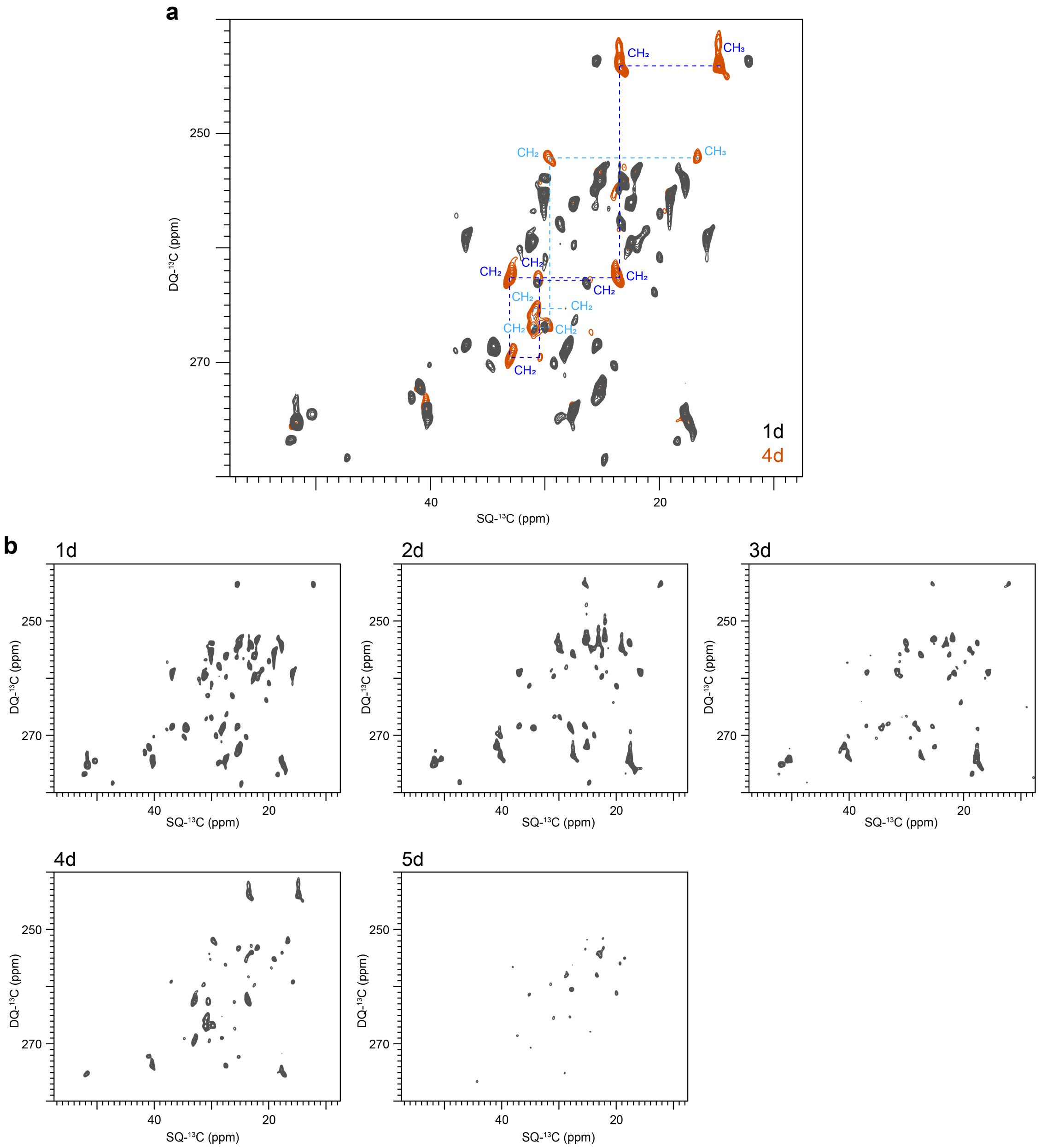

Supplementary Figure 5. Evolution of lipids/biosurfactant signals in the mobile phase. 2D ^{13}C–^{13}C DP *J*-INADEQUATE spectra of biofilm samples resolve the aliphatic carbon signals, likely originating from the lipid acyl chain or biosurfactants. (a) Overlay of spectra from day 1 (black) and day 4 (red) samples. Several prominent peaks of CH_2 and CH_3 groups appear exclusively in the day 4 sample. The potential connectivities were labeled and marked with dash lines. (b) The corresponding spectral region from Day 1 to Day 5.

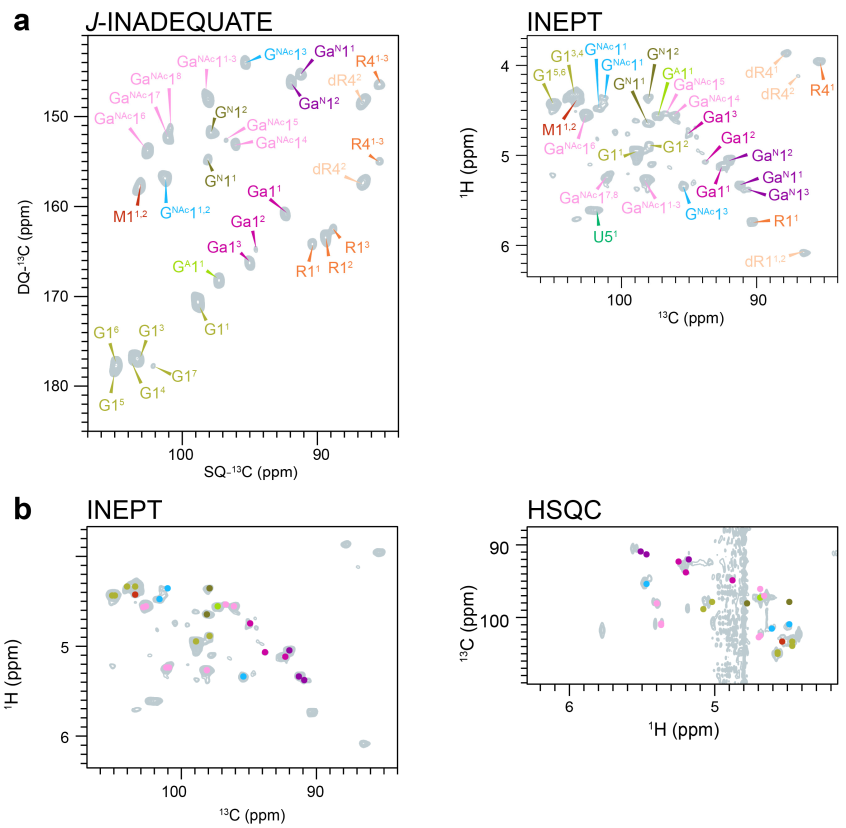

Supplementary Figure 6. Representative anomeric carbon signals of monosaccharide compositions detected across various types of 2D NMR spectra. (a) 2D ^{13}C–^{13}C DP *J*-INADEQUATE and ^{13}C–^{1}H INEPT spectra detect mobile and highly mobile carbohydrates within *B. subtilis* biofilm, respectively. The detected mobile carbohydrates include a portion of the cell wall carbohydrates and the entire exopolysaccharides. The assignments are labeled with abbreviations and color-coded using the same scheme as in Figure 5. (b) 2D ^{1}H–^{13}C HSQC spectrum detects carbohydrates released into the spent medium. To facilitate mapping of the INEPT signals from the biofilm sample onto the HSQC spectrum of the spent medium, a simulated spectrum was generated from the INEPT data and overlaid on the HSQC spectrum.

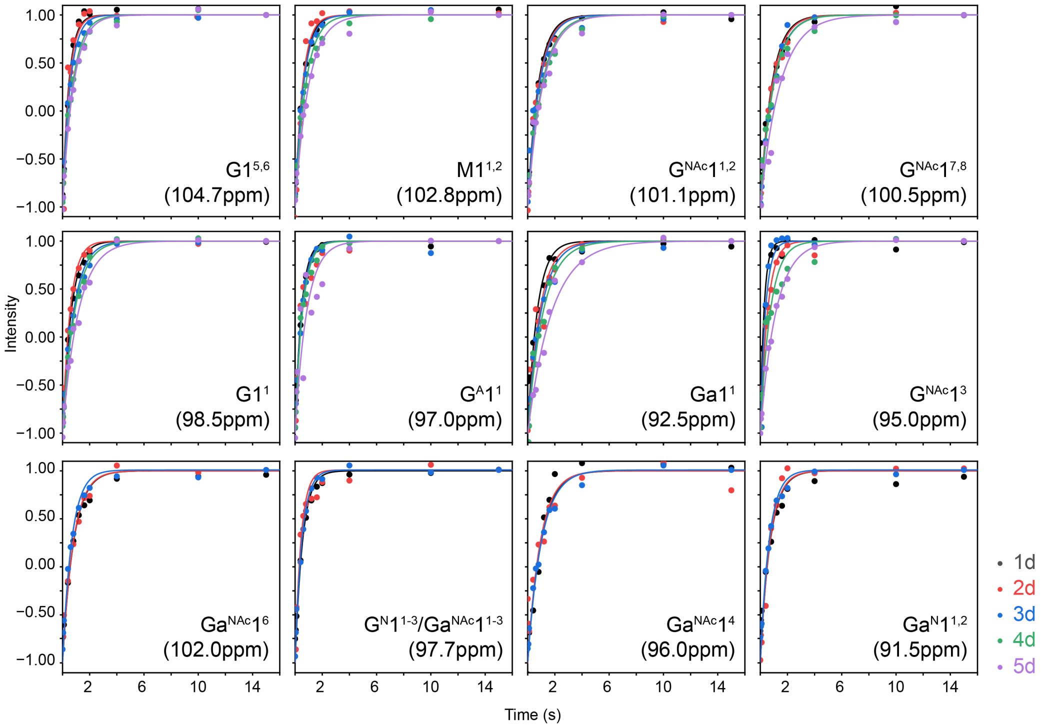

Supplementary Figure 7. Selective ^{13}C-T_1 relaxation curves of exopolysaccharides in *B. subtilis* biofilm.

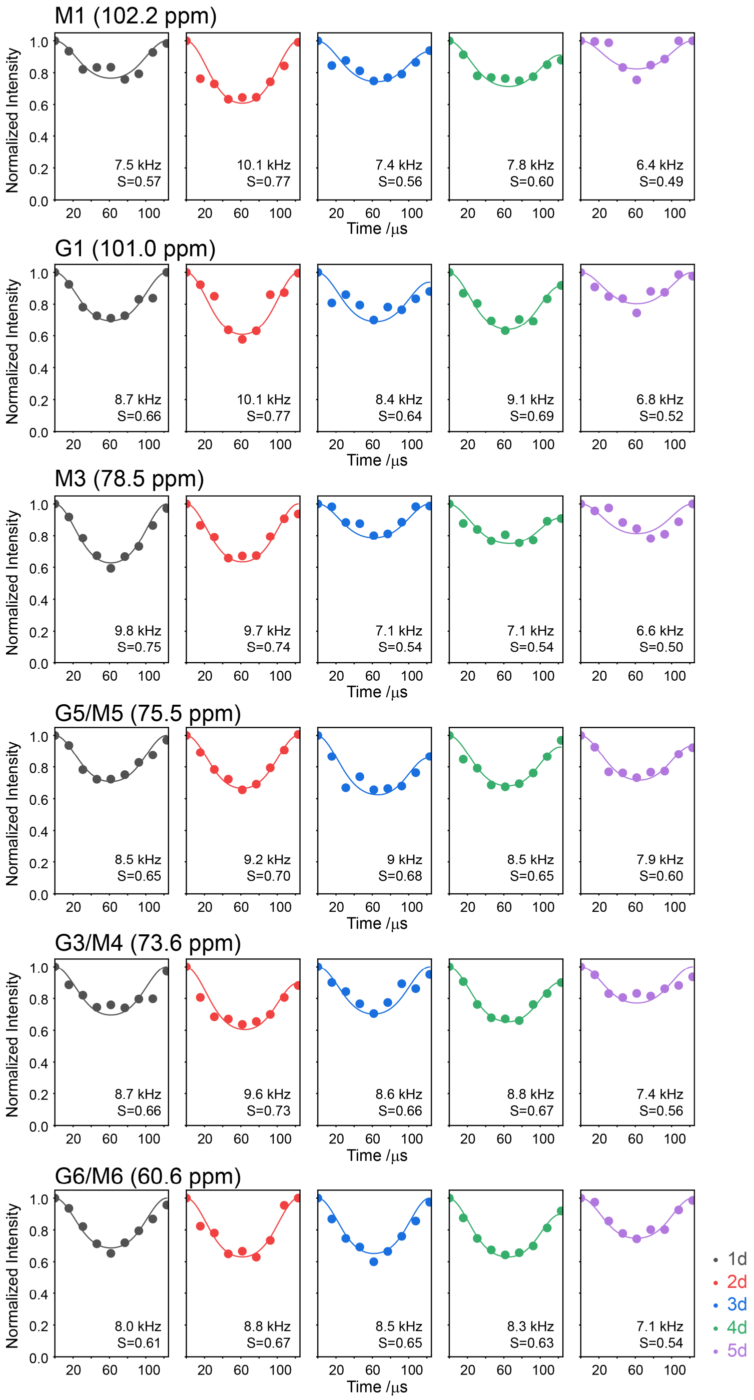

Supplementary Figure 8. DIPSHIFT curves of bacteria cell wall polymers in the rigid phase, derived from 1D CP-DIPSHIFT spectra. The best-fit dipolar couplings, along with the corresponding order parameters are labeled.

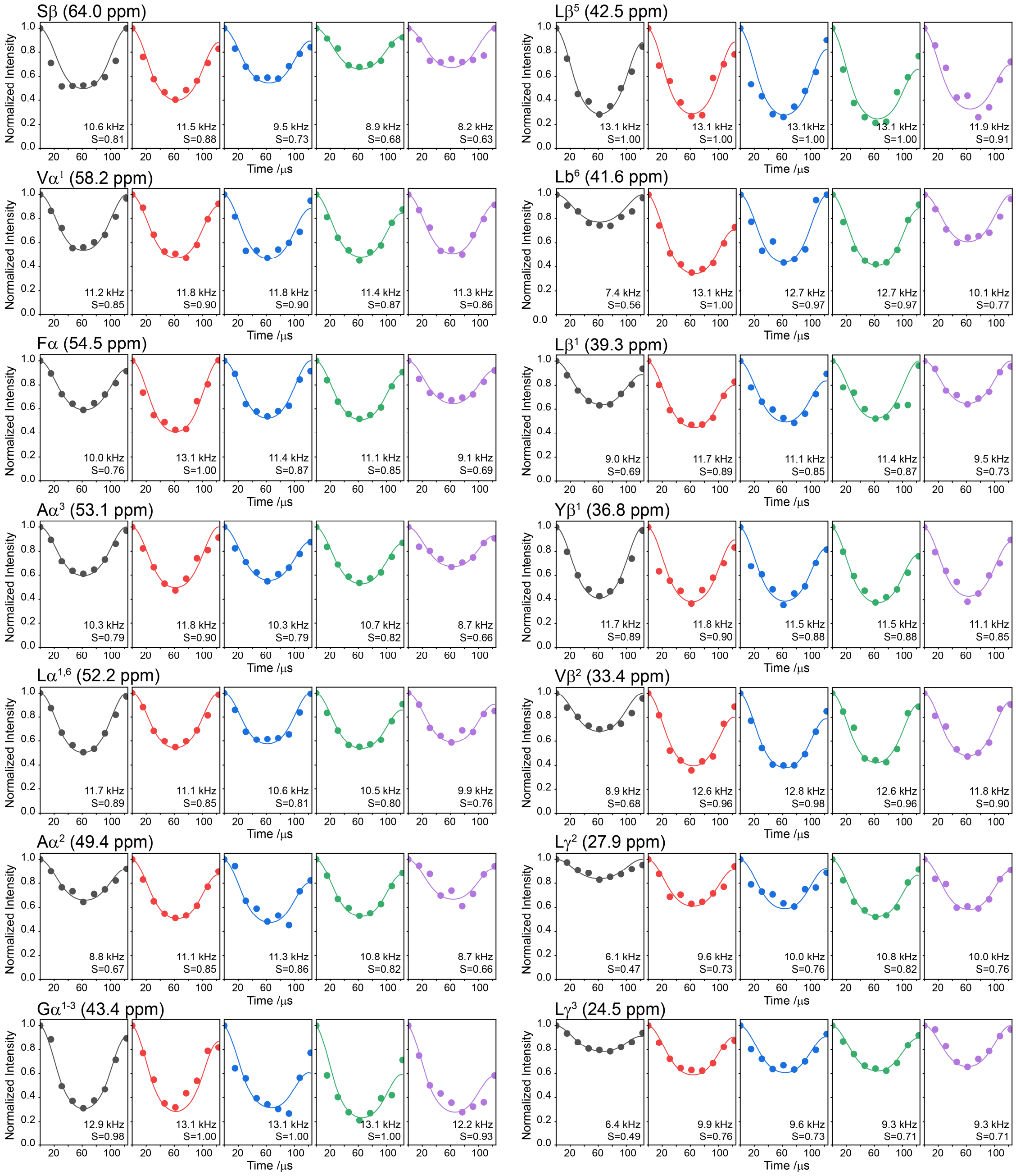

Supplementary Figure 9. DIPSHIFT curves of proteins in the rigid phase, derived from 1D CP-DIPSHIFT spectra. The best-fit dipolar couplings, along with the corresponding order parameters are labeled.

Supplementary Table 1. Glycerol consumption in the spent medium over the 5-day period. The intensity of the signature peak at 62.5 ppm for glycerol is used for quantitative analysis. Error bars are derived from the signal-to-noise ratio of the spectra.

Time (Days)	Peak intensity	NS	Intensity/NS	Normalized
0	1.04E+12	2048	5.08E+08	1.00±0.01
1	1.90E+11	1024	1.86E+08	0.37±0.02
2	2.02E+10	1024	1.97E+07	0.04±0.03
3	2.45E+10	1024	2.39E+07	0.05±0.03
4	2.61E+10	1024	2.55E+07	0.05±0.04
5	9.24E+09	1024	9.02E+06	0.02±0.02

Supplementary Table 2. Data analysis of relative carbon biomass density within *B. subtilis* biofilm. The 1D quantitative integral is proportional to the absolute carbon biomass of the measured sample. To enable comparisons among samples with varying sample weights and differing numbers of scans (NS) during data collection, a calibrated integral is calculated by normalizing for these factors. The calibrated integral is linearly correlated with the carbon biomass weight percentage of the sample, which we define in this context as 'carbon biomass density' for simplicity. The relative carbon mass density is calculated by normalizing against the maximum values observed in the Day-2 sample. Only data from batch-2 samples were used to generate **Figure 2b**.

**Batch-1**					
Time (Days)	Weight (mg)	1D quantitative DP integral (ABS)	NS	Calibrated integral	Relative carbon biomass density
1	33.6	1.3334E+13	512	7.7509E+08	89.1%
2	31.3	3.4859E+12	128	8.7007E+08	100.0%
3	34.6	9.1219E+12	512	5.1492E+08	59.2%
4	31.6	3.5547E+12	512	2.1971E+08	25.3%
5	33.4	3.1848E+12	512	1.8624E+08	21.4%
**Batch-2**					
Time (Days)	Weight (mg)	1D quantitative DP integral (ABS)	NS	Calibrated integral	Relative carbon biomass density
1	33.1	1.3266E+13	512	7.8279E+08	81.1%
2	34.4	1.6991E+13	512	9.6468E+08	100.0%
3	32.4	1.6281E+13	1024	4.9071E+08	50.9%
4	33.4	4.1905E+12	512	2.4505E+08	25.4%
5	32.4	3.1851E+12	512	1.9200E+08	19.9%

Supplementary Table 3. Data analysis of mobile phase proportion within *B. subtilis* biofilm. The proportion of mobile phase is calculated by comparing the total integral of the 1D ^{13}C 2s DP spectrum with that of the 1D ^{13}C quantitative DP spectra for two batches of samples. Calibration of NS is required to align the two spectra. Only data from batch-2 samples were used to generate the **Figure 2d**.

**Batch-1**							
Time (Days)	1D quantitative DP integral (ABS)	NS (quantitative DP)	1D 2s DP integral (ABS)	NS (2s DP)	quantitative DP integral/NS	2s DP integral/NS	Mobile percentage
1	1.3334E+13	512	1.2137E+13	512	2.6043E+10	2.3705E+10	91.0%
2	3.4859E+12	128	2.9956E+12	128	2.7233E+10	2.3403E+10	85.9%
3	9.1219E+12	512	7.2229E+12	512	1.7816E+10	1.4107E+10	79.2%
4	3.5547E+12	512	2.7330E+12	512	6.9427E+09	5.3378E+09	76.9%
5	3.1848E+12	512	2.3818E+12	512	6.2203E+09	4.6519E+09	74.8%
**Batch-2**							
Time (Days)	1D quantitative DP integral (ABS)	NS (quantitative DP)	1D 2s DP integral (ABS)	NS (2s DP)	quantitative DP integral/NS	2s DP integral/NS	Mobile percentage
1	1.3266E+13	512	2.4027E+13	1024	2.5910E+10	2.3464E+10	90.6%
2	1.6991E+13	512	1.5171E+13	512	3.3185E+10	2.9631E+10	89.3%
3	1.6281E+13	1024	2.5108E+13	2048	1.5899E+10	1.2260E+10	77.1%
4	4.1905E+12	512	6.3686E+12	1024	8.1845E+09	6.2193E+09	76.0%
5	3.1851E+12	512	9.6499E+12	2048	6.2209E+09	4.7119E+09	75.7%

Supplementary Table 4. The temporal profiles of carbohydrates and proteins carbon biomass density within *B. subtilis* biofilm. The data are derived from the 1D ^{13}C quantitative spectra by calculating the integrals of selected regions. Calibration of samples weights and NS is required. The value of Day-2 sample is arbitrarily set as the reference (1.000) to facilitate comparison across samples. Only data from batch-2 samples were used in all subsequent supplementary tables.

Time (Days)	Sample Weight (mg)	NS	Carbohydrates integral (ABS) [a]	Carbohydrates calibrated integral	Relative carbon biomass density
1	33.1	512	1.59E+12	9.36E+07	0.776
2	34.4	512	2.12E+12	1.21E+08	1.000
3	32.4	1024	2.89E+12	8.72E+07	0.723
4	33.4	512	6.16E+11	3.60E+07	0.299
5	32.4	512	4.78E+11	2.88E+07	0.237

Time (Days)	Sample Weight (mg)	NS	Proteins integral (ABS) [b]	Proteins calibrated integral	Relative carbon biomass density
1	33.1	512	4.97E+12	2.93E+08	0.849
2	34.4	512	6.08E+12	3.45E+08	1.000
3	32.4	1024	6.32E+12	1.90E+08	0.552
4	33.4	512	1.33E+12	7.76E+07	0.225
5	32.4	512	1.10E+12	6.61E+07	0.191

a. Integral region for carbohydrates: 74-105 ppm.

b. Integral region for proteins: 10-29, 34-48, 106-124, 132-160 ppm.

Supplementary Table 5. Calculation of mobile proportions of carbohydrates and proteins. The mobile proportions of carbohydrates and proteins are computed by comparing peak integrals in specified regions (same as in Supplementary Table 4) between the 2s DP and quantitative DP spectra. Calibration of NS is necessary.

Time (Days)	Carbohydrates calibrated integral (quantitative DP)	Carbohydrates calibrated integral (2s DP)	Mobile carbohydrates proportion (%)
1	9.36E+07	8.83E+07	94.29%
2	1.21E+08	1.13E+08	93.36%
3	8.72E+07	7.34E+07	84.18%
4	3.60E+07	2.64E+07	73.42%
5	2.88E+07	2.04E+07	70.84%

Time (Days)	Proteins calibrated integral (quantitative DP)	Proteins calibrated integral (2s DP)	Mobile proteins proportion (%)
1	2.93E+08	2.56E+08	87.17%
2	3.45E+08	2.90E+08	83.87%
3	1.90E+08	1.44E+08	75.34%
4	7.76E+07	5.81E+07	74.84%
5	6.61E+07	4.86E+07	73.57%

Supplementary Table 6. Chemical shifts of ^{13}C and ^{1}H for anomeric carbon and proton of exopolysaccharides in *B. subtilis* biofilm. Superscripts are used to denote different allomorphs. (-) Undetected. The day(s) on which the subtype is detectable are noted. The newly emerged sugar type is underlined.

Type	C1 (ppm)	H1 (ppm)	Note
$G^{NAc,1}$	100.8	4.36	1d-5d
$G^{NAc,2}$	101.4	4.48	1d, 2d, 4d
$G^{NAc,3}$	95.2	5.34	1d-5d
$\underline{G^{NAc,4}}$	100.0	-	$\underline{\text{4d}}$
M^{1}	103.2	4.43	1d-5d
$\underline{M^{2}}$	$\underline{102.6}$	$\underline{4.43}$	$\underline{\text{5d}}$
G^{1}	98.7	4.95	1d-5d
G^{2}	97.7	4.89	1d-5d, only in HSQC
G^{3}	103.2	4.34	1d-5d
G^{4}	103.8	4.34	1d-5d
G^{5}	104.9	4.44	1d, 2d, 3d
G^{6}	104.7	4.44	1d-5d
G^{7}	102.1	-	1d
$G^{A,1}$	97.1	4.56	1d-5d
G^{N1}	97.9	4.65	1d, 2d
$G^{N,2}$	97.7	4.36	1d
$\underline{G^{N,3}}$	$\underline{98.3}$	-	$\underline{\text{2d}}$
$\underline{G^{N,4}}$	$\underline{99.1}$	-	$\underline{\text{3d}}$
Ga^{1}	92.1	5.12	1d-5d
Ga^{2}	93.6	5.07	1d, 2d
Ga^{3}	94.7	4.75	1d, 2d
$\underline{Ga^{4}}$	$\underline{93.3}$	-	$\underline{\text{2d}}$
$\underline{Ga^{5}}$	$\underline{95.0}$	-	$\underline{\text{4d}}$
$Ga^{N,1}$	91.1	5.34	1d, 2d, 3d
$Ga^{N,2}$	91.8	5.05	1d, 2d, 3d
$Ga^{NAc,1}$	97.9	5.27	1d, 2d, 3d, 4d
$Ga^{NAc,2}$	97.9	5.27	1d, 2d, 3d, 4d
$Ga^{NAc,3}$	97.9	5.27	1d, 2d, 3d, 4d
$Ga^{NAc,4}$	95.9	4.56	1d, 2d, 3d
$Ga^{NAc,5}$	96.5	4.54	1d, 2d, 3d
$Ga^{NAc,6}$	102.4	4.56	1d-5d
$Ga^{NAc,7}$	100.9	5.24	1d, 2d, 3d
$Ga^{NAc,8}$	100.7	5.24	1d, 2d

Supplementary Table 7. Chemical shifts of aliphatic carbons emerged on day 4. Chemical shifts of aliphatic carbons from various biosurfactants, as reported in previous literature using solution NMR, are also listed for comparison.

Type	C (ppm)				
Aliphatic-CH_2 [a]	23.4	29.6	26.0	30.4	33.0
Aliphatic-CH_3 [a]	14.8	16.5			
Aliphatic-CH_2 in *B. subtilis* OKB105 [1]	28.4	34.0			
Aliphatic-CH_3 in *B. subtilis* OKB105 [1]	14.7	11.7	19.0		
Aliphatic-CH_2 in *Bacillus sp.* [2]	24.3	28.4-29.4	26.7	33.6	35.9
Aliphatic-CH_3 in *Bacillus sp.* [2]	11.1	13.8	19.0		
Aliphatic-CH_2 in *B. amyloliquefaciens SH-B74* [3]	22.2	28.8-29.2	25.4	31.4	34.0
Aliphatic-CH_3 in *B. amyloliquefaciens SH-B74* [3]	14.1				
Aliphatic-CH_2 in *B. velezensis T701* [4]	22.3	28.9-29.4	25.6-26.3	30.4	
Aliphatic-CH_3 in *B. velezensis T701* [4]	13.0				

a. Chemical shifts presented in this study.

Supplementary Table 8. Semi-quantification of the mobile molecule phase in *B. subtilis* biofilm. The integral data are derived from 2D *J*-INADEQUATE spectra. The components within the mobile phase are categorized to seven groups: bacteria-related, exopolysaccharides, proteins, nucleotides, N-acetyl group, lipids/biosurfactants and unknown. Bacteria-related group includes cell wall peptidoglycan (G^{NAc}, M, G^{N} end group, D-lactate (Lac), 2,6-Diaminopimelic acid (A_2pm), D-Glu) and wall/lipid teichoic acids (polyglycerol phosphate (pg), G^{NAc} sidechain). Nucleotides group includes ribose (R), deoxyribose (dR) and nucleotide bases (uracil (U)). N-acetyl group includes both methyl group and carbonyl. Lipids/biosurfactants group includes acyl group (CH_2) and methyl group (CH_3). Abbreviations for exopolysaccharides are same as in Figure 4. One-letter amino acid names are used.

	Type	Day 1		Day2		Day3		Day 4		Day 5	
		Avg. integrals	Type%	Avg. integrals	Type%	Avg. integrals	Type%	Avg. integrals	Type%	Avg. integrals	Type%
Bacterial-related	G^{NAc}	2.11E+12	1.49%	6.07E+12	2.58%	5.14E+12	3.14%	2.58E+12	4.05%	2.16E+12	5.50%
	M	1.29E+12	0.91%	3.04E+12	1.29%	2.39E+12	1.46%	2.03E+12	3.18%	1.52E+12	3.87%
	Lac	1.13E+12	0.80%	2.93E+12	1.24%	6.80E+12	4.15%	5.85E+11	0.92%	0.00E+00	0.00%
	A_2pm	5.72E+12	4.04%	5.20E+12	2.21%	7.63E+12	4.66%	1.74E+12	2.74%	3.74E+11	0.95%
	pg	4.36E+11	0.31%	2.51E+12	1.07%	1.58E+12	0.96%	1.43E+12	2.24%	1.36E+12	3.45%
	D-Glu	2.59E+12	1.83%	2.56E+12	1.09%	3.28E+12	2.00%	1.21E+12	1.90%	0.00E+00	0.00%
	G^{N} (end)	6.11E+11	0.43%	1.21E+12	0.51%	8.31E+11	0.51%	0.00E+00	0.00%	0.00E+00	0.00%
Exopolysaccharides	G	2.73E+13	19.32%	4.85E+13	20.62%	2.51E+13	15.36%	1.41E+13	22.09%	1.35E+13	34.20%
	G^{A}	3.22E+12	2.28%	4.55E+12	1.93%	7.24E+12	4.43%	3.57E+12	5.60%	3.27E+12	8.31%
	G^{N}	1.16E+12	0.82%	1.62E+12	0.69%	2.66E+11	0.16%	7.20E+10	0.11%	6.35E+10	0.16%
	Ga	1.94E+12	1.37%	6.96E+12	2.95%	3.96E+12	2.42%	1.89E+12	2.96%	1.22E+12	3.11%
	Ga^{N}	2.61E+12	1.84%	4.53E+12	1.92%	9.06E+12	5.54%	8.33E+10	0.13%	2.83E+10	0.07%
	Ga^{NAc}	8.17E+12	5.78%	8.20E+12	3.48%	9.82E+12	6.00%	2.11E+12	3.31%	9.11E+11	2.31%
Proteins	A	8.08E+12	5.71%	9.40E+12	3.99%	1.18E+13	7.21%	4.16E+12	6.54%	7.55E+11	1.92%
	E	8.65E+12	6.12%	6.00E+12	2.55%	4.16E+12	2.54%	1.40E+12	2.20%	0.00E+00	0.00%
	S	4.29E+11	0.30%	5.85E+11	0.25%	7.93E+11	0.48%	2.85E+11	0.45%	6.10E+11	1.55%
	T	6.30E+12	4.45%	1.65E+13	7.00%	8.94E+12	5.47%	1.27E+12	1.99%	4.63E+12	11.76%
	F	6.46E+12	4.56%	6.76E+12	2.87%	2.96E+12	1.81%	9.66E+11	1.52%	0.00E+00	0.00%
	Y	9.36E+12	6.62%	1.61E+13	6.85%	2.45E+12	1.50%	8.27E+11	1.30%	0.00E+00	0.00%
	W	2.85E+12	2.01%	5.54E+12	2.36%	6.79E+12	4.15%	3.25E+12	5.10%	2.52E+12	6.40%
	G	3.26E+12	2.31%	8.75E+12	3.72%	3.17E+12	1.94%	1.53E+12	2.40%	3.42E+11	0.87%
	V	5.13E+12	3.63%	9.42E+12	4.00%	2.39E+12	1.46%	1.50E+12	2.35%	5.60E+11	1.42%
	I	2.18E+12	1.54%	5.33E+12	2.26%	1.66E+12	1.02%	1.20E+12	1.88%	0.00E+00	0.00%

	L	1.34E+13	9.45%	2.34E+13	9.96%	6.63E+12	4.05%	4.18E+12	6.57%	1.78E+12	4.51%
	N	7.39E+11	0.52%	1.28E+12	0.54%	3.96E+11	0.24%	0.00E+00	0.00%	0.00E+00	0.00%
Nucleotides	U	1.25E+12	0.88%	1.96E+12	0.83%	2.96E+11	0.18%	3.16E+11	0.50%	0.00E+00	0.00%
	dR	6.69E+11	0.47%	8.28E+11	0.35%	9.74E+11	0.60%	0.00E+00	0.00%	0.00E+00	0.00%
	R	9.78E+11	0.69%	5.76E+11	0.24%	4.86E+11	0.30%	3.67E+11	0.58%	0.00E+00	0.00%
N-acetyl	NAc	5.60E+12	3.96%	9.43E+12	4.01%	1.28E+13	7.83%	4.81E+12	7.55%	2.70E+12	6.85%
Lipids/biosurfactants		2.34E+11	0.17%	4.21E+11	0.18%	2.09E+11	0.13%	1.91E+12	3.00%	0.00E+00	0.00%
Unknown		7.61E+12	5.38%	1.51E+13	6.43%	1.36E+13	8.32%	4.36E+12	6.85%	1.10E+12	2.79%

Supplementary Table 9. Summary of semi-quantitative analysis. Absolute quantity and relative proportion of each group are calculated from data in Supplementary Table 8.

	Total integral (ABS)	Bacterial-related%	Exopolysaccharides%	Proteins%	Nucleotides%	N-acetyl%	Lipids /biosurfactants %	Unknown%
Day 1	1.41E+14	9.82	31.41	47.22	2.05	3.96	0.17	5.38
Day 2	2.35E+14	10.00	31.60	46.36	1.43	4.01	0.18	6.42
Day 3	1.64E+14	16.89	33.90	31.85	1.07	7.83	0.13	8.32
Day 4	6.37E+13	15.03	34.20	32.29	1.08	7.55	3.00	6.85
Day 5	3.94E+13	13.77	48.16	28.43	0.00	6.85	0.00	2.79

	Total integral (ABS)	Samples weight (mg)	Integral per mg samples	Bacterial-related	Exopolysaccharides	Proteins	Nucleotide	N-acetyl	Lipids /biosurfactants	Unknown
Day 1	1.41E+14	33.1	4.27E+12	4.20E+11	1.34E+12	2.02E+12	8.76E+10	1.69E+11	7.27E+09	2.30E+11
Day 2	2.35E+14	34.4	6.84E+12	6.84E+11	2.16E+12	3.17E+12	9.78E+10	2.74E+11	1.23E+10	4.39E+11
Day 3	1.64E+14	32.4	5.05E+12	8.53E+11	1.71E+12	1.61E+12	5.40E+10	3.96E+11	6.57E+09	4.20E+11
Day 4	6.37E+13	33.4	1.91E+12	2.87E+11	6.52E+11	6.16E+11	2.06E+10	1.44E+11	5.72E+10	1.31E+11
Day 5	3.94E+13	32.4	1.21E+12	1.67E+11	5.85E+11	3.45E+11	0.00E+00	8.32E+10	0.00E+00	3.39E+10

	Bacterial-related	Exopolysaccharides	Proteins	Nucleotide	N-acetyl	Lipids /biosurfactants	Unknown
Day 1	13.23	42.32	63.63	2.76	5.34	0.23	7.25
Day 2	21.57	68.16	100.00	3.08	8.65	0.39	13.85
Day 3	26.90	53.98	50.72	1.70	12.47	0.21	13.25
Day 4	9.03	20.55	19.41	0.65	4.54	1.80	4.12
Day 5	5.27	18.44	10.89	0.00	2.62	0.00	1.07

Supplementary Table 10. Monosaccharide compositions within biofilm. The integrals are derived from in 2D ^{13}C–^{13}C *J*-INADEQUATE spectra (**Supplementary Table 7**).

	Samples weight (mg)	G^{NAc} (pg) [a]	M	G	G^{A}	G^{N}	Ga	Ga^{N}	Ga^{NAc}
Day 1	33.1	1.24E+12	1.29E+12	2.73E+13	3.22E+12	1.78E+12	1.94E+12	2.61E+12	8.17E+12
Day 2	34.3	2.81E+12	3.04E+12	4.85E+13	4.55E+12	2.83E+12	6.96E+12	4.53E+12	8.20E+12
Day 3	32.4	2.05E+12	2.39E+12	2.51E+13	7.24E+12	1.10E+12	3.96E+12	9.06E+12	9.82E+12
Day 4	33.4	1.99E+12	2.03E+12	1.41E+13	3.57E+12	7.20E+10	1.89E+12	8.33E+10	2.11E+12
Day 5	32.4	1.16E+12	1.52E+12	1.35E+13	3.27E+12	6.35E+10	1.22E+12	2.83E+10	9.11E+11

	G^{NAc} (pg)	M	G	G^{A}	G^{N}	Ga	Ga^{N}	Ga^{NAc}
Day 1	3.76E+10	3.90E+10	8.26E+11	9.73E+10	5.36E+10	5.85E+10	7.88E+10	2.47E+11
Day 2	8.18E+10	8.87E+10	1.42E+12	1.33E+11	8.24E+10	2.03E+11	1.32E+11	2.39E+11
Day 3	6.33E+10	7.38E+10	7.76E+11	2.24E+11	3.39E+10	1.22E+11	2.80E+11	3.03E+11
Day 4	5.97E+10	6.07E+10	4.21E+11	1.07E+11	2.16E+09	5.65E+10	2.49E+09	6.31E+10
Day 5	3.58E+10	4.70E+10	4.15E+11	1.01E+11	1.96E+09	3.77E+10	8.74E+08	2.81E+10

	G^{NAc} (pg)	M	G	G^{A}	G^{N}	Ga	Ga^{N}	Ga^{NAc}
Day 1	45.95%	43.98%	58.36%	43.52%	65.08%	28.86%	28.18%	81.43%
Day 2	100.00%	100.00%	100.00%	59.35%	100.00%	100.00%	47.23%	78.82%
Day 3	77.43%	83.29%	54.82%	100.00%	41.07%	60.25%	100.00%	100.00%
Day 4	73.00%	68.42%	29.76%	47.76%	2.62%	27.87%	0.89%	20.80%
Day 5	43.82%	52.98%	29.36%	45.18%	2.38%	18.61%	0.31%	9.27%

a. G^{NAc} (pg) denotes the subtype in peptidoglycan chain.

Supplementary Table 11. Monosaccharide compositions within medium. The amounts of monosaccharides in the medium are estimated by integrating the C1–H1 cross-peaks in 2D ^{1}H–^{13}C HSQC spectra.

	G^{NAc} (pg) [a]	M	G	G^{A}	Ga	Ga^{N}	Ga^{NAc}
Day 1	8.55E+10	1.04E+11	1.02E+12	-	4.77E+12	1.65E+12	5.72E+12
Day 2	9.98E+10	1.06E+11	1.04E+12	4.45E+11	5.03E+12	2.84E+12	6.03E+12
Day 3	1.77E+11	2.13E+11	1.90E+12	4.89E+11	6.38E+12	3.28E+12	7.10E+12
Day 4	2.12E+11	2.40E+11	1.84E+12	5.02E+11	5.30E+12	3.02E+12	6.73E+12
Day 5	2.16E+11	3.01E+11	2.35E+12	5.47E+11	3.17E+12	1.44E+12	4.11E+12

	G^{NAc} (pg)	M	G	G^{A}	Ga	Ga^{N}	Ga^{NAc}
Day 1	39.59%	34.59%	43.63%	-	74.78%	50.36%	80.57%
Day 2	46.23%	35.21%	44.34%	81.47%	78.76%	86.41%	84.92%
Day 3	82.08%	71.01%	80.75%	89.52%	100.00%	100.00%	100.00%
Day 4	98.24%	79.80%	78.31%	91.88%	83.05%	92.07%	94.90%
Day 5	100.00%	100.00%	100.00%	100.00%	49.59%	43.92%	57.87%

a. G^{NAc} (pg) denotes the subtype in peptidoglycan chain.

Supplementary Table 12. Raw data and calibrated data in principle component analysis.

Biofilm	G^{NAc} (pg) [a]	M	G	G^{A}	Ga	Ga^{N}	Ga^{NAc}
Day 2	1.36E+10	1.48E+10	2.36E+11	2.21E+10	3.38E+10	2.20E+10	3.98E+10
Day 3	1.06E+10	1.23E+10	1.29E+11	3.73E+10	2.04E+10	4.66E+10	5.05E+10
Day 4	9.95E+09	1.01E+10	7.02E+10	1.78E+10	9.42E+09	4.16E+08	1.05E+10
Day 5	5.97E+09	7.83E+09	6.92E+10	1.68E+10	6.29E+09	1.46E+08	4.68E+09

Medium	G^{NAC} (pg)	M	G	G^{A}	Ga	Ga^{N}	Ga^{NAc}
Day 2	9.98E+10	1.06E+11	1.04E+12	4.45E+11	5.03E+12	2.84E+12	6.03E+12
Day 3	1.77E+11	2.13E+11	1.90E+12	4.89E+11	6.38E+12	3.28E+12	7.10E+12
Day 4	2.12E+11	2.40E+11	1.84E+12	5.02E+11	5.30E+12	3.02E+12	6.73E+12
Day 5	2.16E+11	3.01E+11	2.35E+12	5.47E+11	3.17E+12	1.44E+12	4.11E+12

$R_{biofilm/medium}$	Glc^{NAc} (pg)	M	G	G^{A}	Ga	Ga^{N}	Ga^{NAc}
Day 2	1.37E-01	1.40E-01	2.27E-01	4.97E-02	6.72E-03	7.76E-03	6.61E-03
Day 3	5.96E-02	5.77E-02	6.82E-02	7.61E-02	3.19E-03	1.42E-02	7.12E-03
Day 4	4.69E-02	4.21E-02	3.82E-02	3.54E-02	1.78E-03	1.38E-04	1.56E-03
Day 5	2.77E-02	2.60E-02	2.95E-02	3.08E-02	1.99E-03	1.01E-04	1.14E-03

$R_{standarized, biofilm/medium}$	Glc^{NAc} (pg)	M	G	G^{A}	Ga	Ga^{N}	Ga^{NAc}
Day 2	0.681520	0.718206	1.801653	-0.40214	-0.93691	-0.92400	-0.93833
Day 3	0.645149	0.578404	0.941923	1.215579	-1.29858	-0.91933	-1.16314
Day 4	1.169788	0.928094	0.728020	0.589604	-1.10732	-1.18998	-1.11821
Day 5	0.800857	0.680734	0.933674	1.029172	-1.08146	-1.21957	-1.14341

a. G^{NAc} (pg) denotes the subtype in peptidoglycan chain.

Supplementary Table 13. ^{13}C-T_1 relaxation time constants of carbohydrates in *B. subtilis* biofilm. The data were measured using standards inversion recovery method with a recycle delay of 15 s. The data are fit using the single exponential equation: $I(t) = e^{-t/T_1}$. Error bars are obtained from the standard deviations of the fitting process.

Assign.	Carbon Site (ppm)	Day 1	Day 2	Day 3	Day 4	Day 5
$G1^{5,6}$	104.7	0.4601±0.0006	0.49±0.05	0.557±0.001	0.737±0.001	0.8056±0.0009
$M1^{1}$	102.8	0.5864±0.0007	0.548±0.007	0.617±0.002	0.773±0.003	0.993±0.003
$G^{NAc}1^{1,2}$	101.1	0.772±0.005	0.820±0.002	0.859±0.009	1.047±0.004	1.108±0.007
$Ga^{NAc}1^{7,8}$	100.5	0.84±0.02	0.880±0.001	0.966±0.002	0.980±0.007	1.44±0.02
$G1^{1}$	98.5	0.642±0.002	0.5536±0.0009	0.815±0.003	0.896±0.001	1.172±0.003
$G^{A}1^{1}$	97.0	0.4601±0.0008	0.500±0.003	0.5002±0.0009	0.543±0.001	0.85±0.03
$Ga1^{1\text{-}3}$	92.5	0.70±0.01	0.89±0.01	0.979±0.007	1.14±0.01	1.78±0.05
$G^{NAc}1^{3}$	95.0	0.273±0.003	0.535±0.001	0.380±0.004	0.733±0.007	1.150±0.001
$Ga^{NAc}1^{6}$	102.0	0.818±0.005	0.802±0.006	0.678±0.002	/	/
$G^{N}1^{1\text{-}3}/Ga^{NAc}1^{1\text{-}3}$	97.7	0.546±0.002	0.431±0.002	0.5002±0.0008	/	/
$Ga^{NAc}1^{4}$	96.0	0.96±0.02	0.935±0.003	1.022±0.004	/	/
$Ga^{N}1^{1,2}$	91.5	0.73±0.01	0.714±0.004	0.652±0.003	/	/

Supplementary Table 14. Dipolar order parameter of rigid compositions in *B. subtilis* biofilm. DC is the best-fit CH or CH_2 dipolar coupling value; S/S_0 is the order parameter calculated by dividing the simulated DC value by the FSLG-scaled rigid-limit CH DC value (13.1 kHz) [5,6]; T_2 represents the T_2 relaxation time used in simulation to correct the asymmetry of the curves. The signals are grouped to: C, carbohydrates; P, proteins; L, lipids. (/) is used for not applicable.

Type	Assign.	Carbon Site (ppm)	Day1			Day2			Day3			Day4			Day5		
			DC (kHz)	S/S_0	T_2 (ms)	DC (kHz)	S/S_0	T_2 (ms)	DC (kHz)	S/S_0	T_2 (ms)	DC (kHz)	S/S_0	T_2 (ms)	DC (kHz)	S/S_0	T_2 (ms)
C	M1	102.2	7.5	0.57	/	10.1	0.77	/	7.4	0.56	1.80	7.8	0.60	1.36	6.4	0.49	/
	G1	101.0	8.7	0.66	/	10.1	0.77	/	8.4	0.64	1.98	9.1	0.69	1.40	6.8	0.52	/
	M3	78.5	9.8	0.75	/	9.7	0.74	/	7.1	0.54	/	7.1	0.54	1.35	6.6	0.50	/
	G5/M5	75.5	8.5	0.65	/	9.2	0.70	/	9.0	0.69	0.82	8.5	0.65	1.68	7.9	0.60	1.84
	G3/M4	73.6	8.7	0.66	/	9.6	0.73	1.15	8.6	0.66	/	8.8	0.67	1.22	7.4	0.56	/
	G6/M6	60.6	8.0	0.61	/	8.8	0.67	/	8.5	0.65	/	8.3	0.63	1.18	7.1	0.54	/
P	Sβ	64.0	10.6	0.81	/	11.5	0.88	0.99	9.5	0.73	1.11	8.9	0.68	1.92	8.2	0.63	/
	$V\alpha^{1}$	58.2	11.2	0.85	/	11.8	0.90	1.28	11.8	0.90	1.03	11.4	0.87	0.75	11.3	0.86	1.50
	Fα	54.5	10.0	0.76	1.74	13.1	1.00	/	11.4	0.87	/	11.1	0.85	1.06	9.1	0.69	1.54
	$A\alpha^{3}$	53.1	10.3	0.79	/	11.8	0.90	/	10.3	0.79	0.98	10.7	0.82	0.85	8.7	0.66	1.67
	$L\alpha^{1,6}$	52.2	11.7	0.89	/	11.1	0.85	/	10.6	0.81	/	10.5	0.80	0.83	9.9	0.76	1.26
	$A\alpha^{2}$	49.4	8.8	0.67	1.5	11.1	0.85	0.91	11.3	0.86	0.58	10.8	0.82	0.95	8.7	0.66	1.63
	$G\alpha^{1\text{-}3}$	43.4	12.9	0.98	1.41	13.1	1.00	0.91	13.1	1.00	0.25	13.1	1.00	0.24	12.2	0.93	0.23
	$L\beta^{5}$	42.5	13.1	1.00	1.00	13.1	1.00	1.04	13.1	1.00	0.65	13.1	1.00	0.30	11.9	0.91	0.35
	$L\beta^{6}$	41.6	7.4	0.56	/	13.1	1.00	0.36	12.7	0.97	/	12.7	0.97	1.04	10.1	0.77	/
	$L\beta^{1}$	39.3	9.0	0.69	1.07	11.7	0.89	0.56	11.1	0.85	0.71	11.4	0.87	/	9.5	0.73	/
	$Y\beta^{1}$	36.8	11.7	0.89	/	11.8	0.90	1.16	11.5	0.88	0.67	11.5	0.88	0.50	11.1	0.85	0.96
	$V\beta^{2}$	33.8	8.9	0.68	/	12.6	0.96	0.57	12.8	0.98	0.53	12.6	0.96	1.33	11.8	0.90	1.76
	$L\gamma^{2}$	27.9	6.1	0.47	/	9.6	0.73	1.41	10.0	0.76	1.61	10.8	0.82	0.89	10.0	0.76	1.24
	$L\gamma^{3}$	24.5	6.4	0.49	1.37	9.9	0.76	1.29	9.6	0.73	1.33	9.3	0.71	116	9.3	0.71	/
L	$-CH_2-$	30.0	4.1	0.31	/	6.0	0.46	/	7.3	0.56	2.42	7.0	0.53	2.2	7.1	0.54	0.74

Supplementary Table 15. NMR experimental details in this work. υ_{MAS} : MAS frequency; SW (F2 or F1) : spectral width for indirect or direct dimension; AQ (F2 or F1) : acquisition time for indirect or direct dimension; NS : number of scans; τ_{mix} : mixing time if have; d1: recycle delay between scans.

Experiments	υ_{MAS} (kHz)	SW (F2) (ppm)	SW (F1) (ppm)	AQ (F2) (ms)	AQ (F1) (ms)	NS	τ_{mix} (ms)	d1 (s)	Processing parameters
Intact biofilm (Day1-Day5)									
1D ^{13}C CP	13.5	/	355	/	14.3	1024	/	2	GM (-5, 0.1)
1D ^{13}C DP	13.5	/	355	/	19.6	128-2048	/	2 or 15	GM (-5, 0.1)
2D ^{13}C-^{1}H refocused INEPT	13.5	355	12.5	21.5	30	16	/	3.5	GM (-10, 0.05)
2D ^{13}C-^{13}C refocused *J*-INADEQUATE	13.5	355	207	14.3	5	96	1.625×2	2	GM (-15, 0.04)
2D ^{13}C-^{13}C DARR	13.5	355	177.5	14.3	4.2	160-288	50	2	GM (-40, 0.025)
1D ^{13}C-DP-T_1 relaxation	13.5	/	355	/	14.3	128-256	/	0.1 μs-15 s	GM (-10, 0.05)
1D ^{13}C-CP-DIPSHFIT	7.8	/	355	/	16.8	2048	/	2	GM (-10, 0.05)
Medium for biofilm culture (Day0-Day5)									
1D ^{13}C	/	/	236.5	/	458.8	512	/	10	EM (3.0)
2D ^{1}H-^{13}C HSQC	/	11	216	155.6	4.9	32	/	10	QSINE (ssb 3)

References

1 Kowall, M. *et al.* Separation and characterization of Surfactin isoforms produced by Bacillus subtilis OKB 105. *Journal of colloid and interface science* **204**, 1-8 (1998).

2 Tang, J. S. *et al.* Complete assignments of 1H and 13C NMR spectral data of nine surfactin isomers. *Magnetic Resonance in Chemistry* **45**, 792-796 (2007).

3 Ma, Z., Hu, J., Wang, X. & Wang, S. NMR spectroscopic and MS/MS spectrometric characterization of a new lipopeptide antibiotic bacillopeptin B1 produced by a marine sediment-derived Bacillus amyloliquefaciens SH-B74. *The Journal of Antibiotics* **67**, 175-178 (2014).

4 Jiang, J. *et al.* Production, purification and characterization of 'Iturin A-2'a lipopeptide with antitumor activity from Chinese Sauerkraut Bacterium Bacillus velezensis T701. *International Journal of Peptide Research and Therapeutics* **27**, 2135-2147 (2021).

5 Hong, M. Determination of multiple ϕ-torsion angles in proteins by selective and extensive 13C labeling and two-dimensional solid-state NMR. *Journal of Magnetic Resonance* **139**, 389-401 (1999).

6 Hong, M. *et al.* Coupling amplification in 2D MAS NMR and its application to torsion angle determination in peptides. *Journal of Magnetic Resonance* **129**, 85-92 (1997).
